# Temporally dynamic neural correlates of drug cue reactivity, response inhibition, and methamphetamine-related response inhibition in people with methamphetamine use disorder

**DOI:** 10.1038/s41598-022-05619-8

**Published:** 2022-03-04

**Authors:** Sara Jafakesh, Arshiya Sangchooli, Ardalan Aarabi, Mohammad Sadegh Helfroush, Amirhossein Dakhili, Mohammad Ali Oghabian, Kamran Kazemi, Hamed Ekhtiari

**Affiliations:** 1 Department of Electrical and Electronics Engineering, Shiraz University of Technology, Shiraz, Iran; 2 Laureate Institute for Brain Research (LIBR), 6655 South Yale Ave., Tulsa, OK 74136 USA; 3 Iranian National Center for Addiction Studies (INCAS), Tehran University of Medical Science, Tehran, Iran; 4 Faculty of Medicine, University of Picardie Jules Verne, Amiens, France; 5 Laboratory of Functional Neuroscience and Pathologies (LNFP), University Research Center (CURS), University Hospital, Amiens, France; 6 Medical Physics Department, Iran University of Medical Sciences, Tehran, Iran; 7 Medical Physics and Biomedical Engineering Department, Tehran University of Medical Sciences, Tehran, Iran

**Keywords:** Biomedical engineering, Computational neuroscience

## Abstract

Cue-induced drug craving and disinhibition are two essential components of continued drug use and relapse in substance use disorders. While these phenomena develop and interact across time, the temporal dynamics of their underlying neural activity remain under-investigated. To explore these dynamics, an analysis of time-varying activation was applied to fMRI data from 62 men with methamphetamine use disorder in their first weeks of recovery in an abstinence-based treatment program. Using a mixed block-event, factorial cue-reactivity/Go-NoGo task and a sliding window across the task duration, dynamically-activated regions were identified in three linear mixed effects models (LMEs). Habituation to drug cues across time was observed in the superior temporal gyri, amygdalae, left hippocampus, and right precuneus, while response inhibition was associated with the sensitization of temporally-dynamic activations across many regions of the inhibitory frontoparietal network. Methamphetamine-related response inhibition was associated with temporally-dynamic activity in the parahippocampal gyri and right precuneus (corrected p-value < 0.001), which show a declining cue-reactivity contrast and an increasing response inhibition contrast. Overall, the declining craving-related activations (habituation) and increasing inhibition-associated activations (sensitization) during the task duration suggest the gradual recruitment of response inhibitory processes and a concurrent habituation to drug cues in areas with temporally-dynamic methamphetamine-related response inhibition. Furthermore, temporally dynamic cue-reactivity and response inhibition were correlated with behavioral and clinical measures such as the severity of methamphetamine use and craving, impulsivity and inhibitory task performance. This exploratory study demonstrates the time-variance of the neural activations undergirding cue-reactivity, response inhibition, and response inhibition during exposure to drug cues, and suggests a method to assess this dynamic interplay. Analyses that can capture temporal fluctuations in the neural substrates of drug cue-reactivity and response inhibition may prove useful for biomarker development by revealing the rate and pattern of sensitization and habituation processes, and may inform mixed cue-exposure intervention paradigms which could promote habituation to drug cues and sensitization in inhibitory control regions.

## Introduction

The prevalence and health burden of methamphetamine use disorder (MUD) continues to increase globally^1^ and in countries such as the US, where 0.4% of the adult population suffers from methamphetamine use disorder^2^ and methamphetamine-related overdose rates have tripled from 2011 to 2016^3^. This potential crisis is compounded by the fact that despite decades of research, assessments of MUD are still largely reliant on clinical interviews^4,5^ and data on effective interventions for MUD remains inconsistent^6^, with growing calls to better delineate the neurobiology of MUD to identify novel treatment targets and clinically-relevant biomarkers^7^. In tandem with research elucidating the involvement of a plethora of cognitive functions in the MUD^8^, functional magnetic resonance imaging (fMRI) studies on the neurobiology of MUD have characterized several functional brain changes associated with cognitive alterations in the MUD, methamphetamine craving, use history and relapse risk, and treatment outcomes^9,10^.

Two central aspects of methamphetamine use disorder are a characteristic reactivity to drug cues (itself involving attentional bias towards drug cues, their increased salience, and ultimately the induction of craving)^11,12^, and failures of executive control and response inhibition^13^. These phenomena widely figure in models of a substance use disorder, such as the “impaired response inhibition and salience attribution” model^14^ and dual-process models, which comprise automatic approach behavior towards substances and reduced abilities to inhibit these behaviors^15^. An increasing number of task-based fMRI studies in MUD have investigated the neural correlates of either methamphetamine cue-reactivity, using conventional cue exposure tasks^16–19^ or cognitive control and response inhibition, using varieties of Stroop, Stop Signal or Go-NoGo tasks^20–22^, but an approach with greater ecological validity has been to assess response inhibition concurrently with cue-reactivity. These latter studies are motivated by the interrelationship between response inhibition and cue-reactivity, with drug cue exposure hampering inhibitory control^23–25^ and poor inhibitory control precipitating higher induced craving^26^. This line of fMRI research typically involves mixed tasks to investigate the response inhibition in the context of exposure to drug cues, such as Go-NoGo tasks in which Go and NoGo signals are independently mixed through neutral and substance cues^27,28^ or in which substance cues are themselves the NoGo signal^29,30^.

A notable characteristic of the above-mentioned studies is an assumption of time-invariant voxel-wise or regional activation, whereby in both response inhibition and cue-reactivity tasks average responses are obtained across the entire task duration. This conventional “static” approach might be problematic in light of the evidence that activation patterns during exposure to emotionally salient stimuli are often dynamic and vary across the task duration, for example when brain regions demonstrate various patterns of habituation to emotionally negative cues or reward stimuli^31–34^ which might be explained through extinction learning^35^, though regional sensitization to pictures of angry faces has also been observed^36^. Considering the potentially multiphasic nature of the cue-reactivity process which unfolds over seconds and minutes and primarily involves different regions and networks at each stage^11^, it’s not surprising that three recent studies on the MUD and opioid use disorder have found evidence of temporally dynamic activation patterns during cue-reactivity in regions such as the amygdala, the dorsal anterior cingulate cortex, the ventromedial prefrontal cortex, the ventral striatum, the caudate nuclei and insular cortices, and various prefrontal regions^37–39^. Given the temporally dynamic involvement of regions such as the bilateral motor and prefrontal cortices in response inhibition^40^ and the dynamic reconfiguration of functional brain networks^41^, it’s reasonable to expect similarly dynamic activation patterns to be implicated in successful and dysfunctional response inhibition in individuals with substance use disorders.

Using data obtained from the first fMRI implementation of a novel mixed cue-reactivity/Go-NoGo task, this study aims to investigate temporally dynamic brain activation patterns that underlies methamphetamine cue-reactivity, response inhibition, and methamphetamine-related response inhibition (response inhibition during exposure to methamphetamine cues) in individuals with MUD. These activation slopes may reflect the sensitization (positive slope) or habituation (negative slope) of brain regions as they engage in response inhibition and the processing of methamphetamine cues. Further, the correlation of activation slopes with clinical and behavioral variables will be investigated, providing an initial estimate of the potential clinical utility of activation slopes.

## Materials and methods

### Participants

Sixty-two men with MUD (age 32.12 ± 5.89) were recruited from addiction treatment centers in Tehran, Iran. Inclusion criteria were (1) Diagnosis of methamphetamine dependence (for at least 6 months) according to the Diagnostic and Statistical Manual of Mental Disorders, Fourth Edition criteria (DSM-IV TR)^42^, (2) abstinence from any substance for at least one week, with the exception of nicotine, based on self-report and confirmed by urine drug screening, (3) right-handedness, determined using the Edinburgh Handedness Inventory^43^, and (4) age between 20 and 40 years. Exclusion Criteria were (a) any comorbid axis‐I disorders other than drug dependence, based on DSM-IV TR criteria, (b) ineligibility for MRI scanning (e.g., metal implants, claustrophobia), (c) history of head trauma resulting in neurological disorders. Nine participants were excluded from fMRI analyses due to excessive movement during scanning, leaving 53 individuals (see “Preprocessing” section for details). Demographic and behavioral data of the 53 participants who were included in the analyses are provided in Table 1.

**Table 1:** Demographic and the profile of male Methamphetamine users (n = 53).

Demographics	Mean (SD)			
Age (years)	32 (5)			
Education (years)	10 (3)			

Psychological assessment	Mean (SD)			
Pre-Scanning Craving Visual Analog Scale (VAS) (0–100)	33 (35)			
Post-Scanning Craving Visual Analog Scale (VAS) (0–100)	30 (37)			
Depression Anxiety and Stress Scale (DASS) (0–60)	27 (14)			
Barratt Impulsiveness Scale, motor score (BIS-Motor) (0–100)	27 (6)			
Barratt Impulsiveness Scale, total score (BIS-Sum) (0–100)	75 (14)			

Drug use profile	Mean (SD)			
Age of Meth use onset	24 (6)			
Meth use duration (years)	7 (7)			
Dosage of Meth (gram per day)	12 (14)			
Cost of Meth (Dollar per month)	467 (391)			

Drug use in the month before treatment	Daily use (%)	Non-daily use (%)	Median of use days for non-daily users	IQR of use days for non-daily users
Methamphetamine	96	4	22	2
Cannabis	49	28	4	5
Alcohol	28	53	2	2
Sedatives	17	13	8	7
Hallucinogens	6	32	2	3
Cocaine	8	19	1	1
Hard opioid	57	4	4	3
Soft opioid	68	15	3	2
Any drug besides methamphetamine	87	6	5	4

Most values are reported as mean (standard deviation). Proportions of daily users (those reporting the use of a substance on every day of the month before entering treatment) and non-daily users (those reporting the use of a substance during the month before treatment, but did not use it on a daily basis) are presented as percentages. For non-daily users, medians and interquartile ranges (IQR) of numbers of use days are provided (for daily users, the median would be 30 and IQR would be 0).

The research protocol was designed and implemented in accordance with the Declaration of Helsinki. After a referral from treatment centers to the research team, individuals were informed regarding the aims of the project, the collected information and measures are taken to ensure anonymity, scanning procedures, the fMRI task and its potential to induce methamphetamine craving, and that they can exit the study at any point with no implications for their ongoing treatment. After the consent form was read both out loud by a psychologist and by the individual to ensure comprehension, participants provided written, informed consent prior to further screening for enrollment. The data collected from each participant was sent to the primary data analyst and anonymized before further processing. The study protocol was reviewed and approved by the ethical review board of the Tehran University of Medical Sciences with the approval code 93-02-98-23869.

### Procedures and measures

Participants were abstinent prior to scanning, but were allowed to smoke. After arriving at the imaging center, participants were interviewed by two clinical psychologists, and several measures were administered prior to scanning. Collected data included demographic information, mental status examination, the Barratt Impulsiveness Scale-11 (BIS-11)^44^, the Depression Anxiety and Stress Scale-21 (DASS-21)^45^, and the Positive and Negative Affect Schedule (PANAS)^46^. The number of days on which participants abused methamphetamine, cannabis, alcohol, sedatives, hallucinogens (including LSD, psilocybin mushrooms and MDMA), cocaine, hard opioids (heroin and crack heroin) and soft opioids (including opium, poppy milk, buprenorphine, diphenoxylate, tramadol and norgesic) in the last 30 days before the start of treatment was assessed using a Drug Abuse Profile instrument. A Risky Behaviors Profile instrument was utilized to assess the history of injecting drug use, high-risk sexual behavior, incarceration, drug selling, and physical altercation.

Methamphetamine craving was assessed using a 0–100 Visual Analog Scale (VAS) before MR scanning, with the question “how much craving are you experiencing right now?”. After scanning, participants were again assessed with PANAS and rated their craving (Table 1). To minimize the risk of drug use after the fMRI session, participants were asked to remain in the scanning center for an hour while recovering.

### fMRI Go-NoGo task

**Figure 1:**
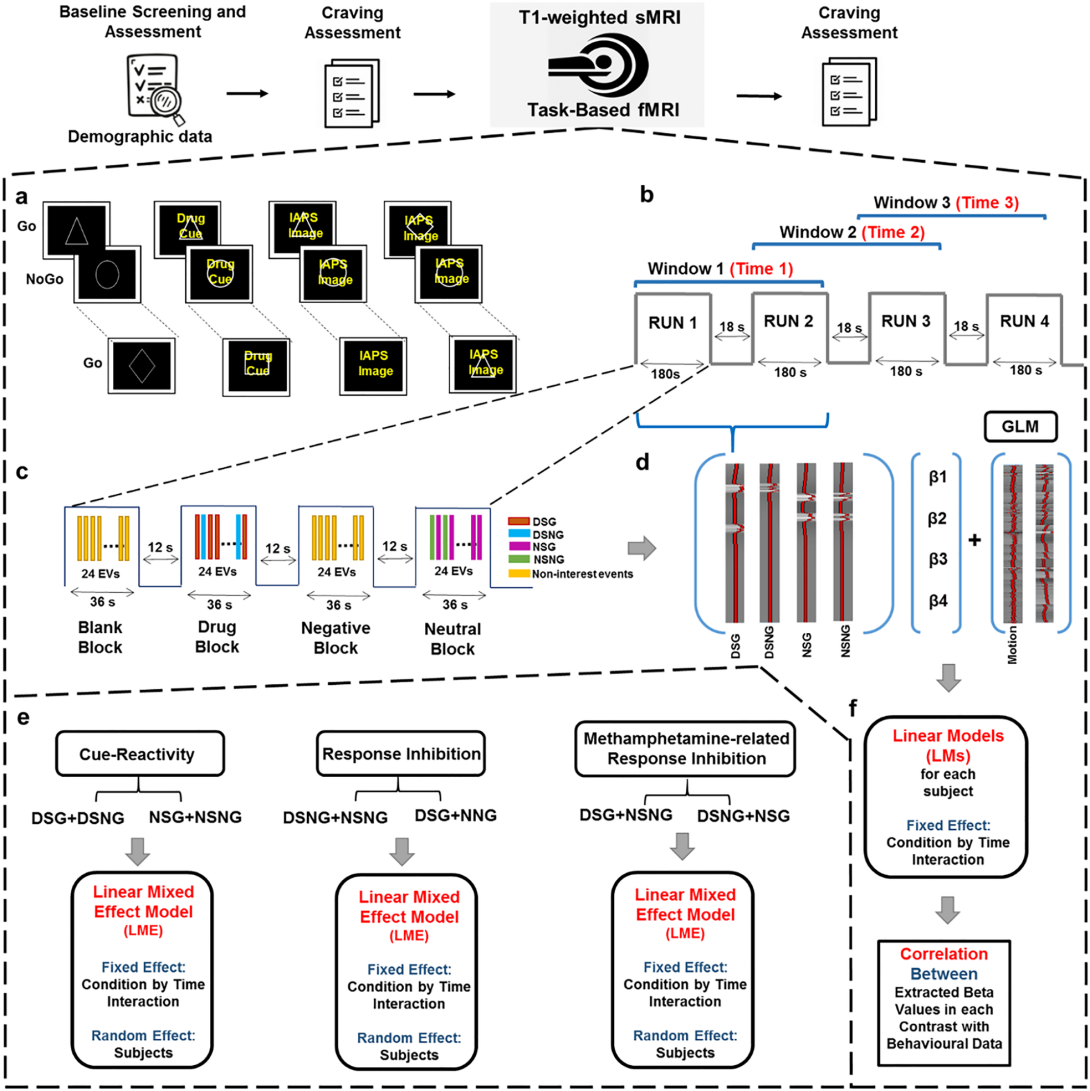
Task procedure. (**a**) Each run contains four blocks with Go-NoGo stimuli superimposed over different backgrounds: Blank, Drug, Neutral, and Negative emotional stimuli (180 s). The blocks are separated by resting blocks with fixation points (18 s). Go stimuli were triangles, squares, and diamonds, and the NoGo stimulus was a circle. Negative and neutral images were chosen from the IAPS database (utilized IAPS pictures are not displayed here due to the IAPS sharing limitations). Drug pictures were obtained from a publicly available and validated cue database (MOCD). (**b**) The task contains four runs and every two consecutive runs are considered a window, yielding three overlapping windows. (**c**) Successful Go and NoGo trials during Drug and Neutral blocks were modelled as four separate event types of interest: Drug Successful Go (DSG), Drug Successful NoGo (DSNG), Neutral Successful Go (NSG), and Neutral Successful NoGo (NSNG). Unsuccessful trials as well as all events on other block types were non interest events. (**d**) In each window, stimulus response functions for the four main event types (DSG, DSNG, NSG, NSNG) entered a General Linear Model (GLM) as regressors of interest. Non interest events were included to design matrix but were not carried forward to further analysis. Head motion and dvars parameters were included as nuisance regressors. (**e**) Beta coefficients for each of 6 conditions estimated in each window are entered in three Linear Mixed Effects Models (LMEs), with random intercepts for subjects and condition-by-time interaction as a fixed effect, to determine regions where coefficients have significant condition by time interactions. (**f**) Subject beta values estimated at the GLM step are entered in linear models for each subject to estimate subject-level interaction slopes at dynamically active regions for each contrast, and the associations between slopes and different clinical and behavioural variables are estimated. Imaging data were analyzed using the fMRI Expert Analysis Tool (FEAT) from FSL (FMRIB’s Software Library, https://fsl.fmrib.ox.ac.uk/fsl) version 6.0.3. Linear Mixed Effect Models and other statistical analyses were conducted using R software version 3.6.2 (https://www.r-project.org/).

Participants were scanned during four consecutive runs of the mixed Go-NoGo task, separated by resting blocks with a fixation point. Each run included four 36-s blocks of 24 stimuli, depicting geometric Go-NoGo signs overlaid on background cues. Background images were either blank (black), neutral images, negative emotional cues, or methamphetamine-related cues. Each block contained 18 Go signs (triangles, squares, or diamonds) and 6 NoGo signs (circles). Each stimulus lasted one second and was followed by a jittered inter-stimulus interval generated using a gamma probability density function (mean = 0.5) (Fig. 1a). The run were separated by 18-s fixation periods in which a white cross was shown on a black background, so each run took 198 s. A total of 16 blocks were presented, four of each condition (blank, neutral, negative, drug). The total scanning duration was approximately 13 min (Fig. 1b).

Participants were instructed to respond as fast as possible when the Go stimuli were presented and to withhold their response to NoGo stimuli. Participants underwent a training test outside the scanner and were informed that both speed and accuracy are important. The methamphetamine cues were obtained from a publicly available and validated cue database^47^, and neutral and negative emotional cues were selected from the IAPS database^48^. The researchers had permission to use the utilized images. Neutral, methamphetamine, and negative cues were matched in terms of visual complexity, brightness, luminance, and color.

### Scanning parameters

Whole-brain T2* weighted images were acquired in a 3.0 Tesla Siemens (MAGNETOM Trio; Germany) scanner in the Tehran University of Medical Sciences, Iran. Functional scans were collected using a 2D gradient echo EPI sequence, and each volume was comprised of 40 contiguous axial slices (TR = 2.2 s, TE = 30 ms, field of view (FOV) = 192 × 192, in-plane voxel size 3.0 mm × 3.0 mm, slice thickness 3 mm, FA = 90°). The scanning session lasted 806 s. A high-resolution T1-weighted structural image was also acquired for each participant for co-registration during preprocessing and to exclude participants with any structural abnormality. Structural images were acquired through a sagittal T1-weighted magnetization-prepared rapid acquisition (MP-RAGE) sequence with the following parameters: repetition time = 1800 ms, echo time = 3.44 ms, FOV = 256 cm × 256 cm, flip angle = 7°, 1 mm^3^ Voxels.

### Pre-processing

FSL (FMRIB’s Software Library, www.fmrib.ox.ac.uk/fsl) version 6.0.3 was used to preprocess structural and functional data^49^. Structural data was skull-stripped to remove non-brain tissue from the structural T1-weighted images using the Brain Extraction Tool (BET). BET parameters were chosen based on each individual skull size.

Functional data were analyzed using the fMRI Expert Analysis Tool (FEAT), part of FMRIB’s Software Library. The functional pre-processing included the removal of the first five volumes, motion correction with 6 degrees of freedom, interleaved slice-timing correction, linear Boundary-Based Registration (*BBR*) of functional images to the high-resolution T1 images, nonlinear registration of the T1 images to the standard Montreal Neurological Institute (MNI) space with 12 degrees of freedom, intensity normalization, smoothing with a 5-mm full-width at half-maximum (FWHM) Gaussian kernel, denoising with melodic ICA, high-pass temporal filtering (with the cut-off frequency equal to the inverse of 120 s).

High motion effects on fMRI time series were identified using the DVARS metric^50^ and were regressed out in the first-level generalized linear model (GLM) analysis. “High movement subjects” were defined as those with displacement > 4 mm and also DVARS > 75 in more than ten volumes in a single block (36 s), and were excluded from the analyses (9 subjects).

### Temporally dynamic fMRI analysis

The data used in this study have been analyzed in a conventional GLM framework before. (Dakhile et al., 2021, ).

In the dynamic analysis, ROI-based whole-brain analyses were performed using the Brainnetome atlas (BNA)^51^. First, the whole brain was parcellated into 246 regions based on the BNA. The BNA masks in MNI space were then registered to each subject’s space using the transformation matrices derived from the pre-processing step and after determining subject-specific masks for each ROI across the 53 subjects, the mean activations and standard errors were calculated. We then used a sliding window over each subject’s fMRI time series, separating the task duration into three overlapping windows with a window duration of two task runs (396 s) and a sliding interval equal to one run (198 s) (Fig. 1b). We used the overlapping window to smooth the transition between windows and have a higher signal-to-noise ratio to increase the power of the LMEs and detect stable dynamically activated regions. An event-related approach was adopted for each window despite the use of drug and neutral cues in blocks, since using boxcars to model block-level activity would lead to the overlapping of cue-reactivity and response inhibition stimulus response functions and could contribute to signal misattribution^52^. The four distinct event types of interest included Neutral Successful NoGo (NSNG), Neutral Successful Go (NSG), Drug Successful NoGo (DSNG) and Drug Successful Go (DSG) events (Fig. 1c). A GLM model was estimated in each window for each subject, with the four event types of interest modelled by four event onset functions and convolved with the canonical hemodynamic response function to obtain four regressors of interest. In each of the three GLM models fit (one per window), Unsuccessful Go and NoGo trials, blank and negative-emotional successful Go and NoGo trials were included as independent no-interest regressors, and their coefficients were not used in further analyses. Six head motion parameters and high motion time-points extracted based on the DVARS metric were included as nuisance regressors (Fig. 1d).

To determine the time-variable neural correlates of methamphetamine cue-reactivity, response inhibition and methamphetamine-related response inhibition, three pairs of conditions were defined using six different combinations of the four regressors. The pairs included cue-reactivity conditions (Drug and Neutral cues), response inhibition conditions (NoGo and Go trials), and methamphetamine-related response inhibition conditions (response inhibition during exposure to either methamphetamine or neutral cues). Using three GLM models, beta coefficients for each condition (each one of the two conditions in a contrast) in each window were estimated separately (Fig. 1e). Overall, 18 beta coefficients per subject were estimated for the 6 conditions and at each brain region over the three windows. The six conditions were defined as Drug (*DSNG* + *DSG*), Neutral (*NSNG* + *NSG*)*,* NoGo (*DSNG* + *NSNG*), Go (*DSG* + *NSG*)*,* response inhibition during methamphetamine cue exposure (*DSNG − DSG*) and response inhibition during neutral cue exposure (*NSNG* − *NSG*). For each of the three pairs of conditions, “dynamically-activated brain regions” were defined as those regions in which the activations associated with the two conditions across time have differing slopes. These regions were identified on the group level by fitting three Linear Mixed Effects (LME) models to subject beta coefficients in each region in R, version 3.6.2^53^ (Fig. 1e). The three models were specified as:

1 $${\varvec{\upbeta}}={\upbeta }_{0}\times (1\left|subject\right)+{\upbeta }_{1}\times time+{\upbeta }_{2}\times condition+{\upbeta }_{3}\times time{:} \; condition+\upepsilon,$$

where ${\varvec{\upbeta}}$ is a merged vector of the 6 sets of beta coefficients associated with either of a pair of conditions over the three windows across all participants, and is estimated as a linear combination of time, condition, and their interaction. To avoid confusion, the coefficients derived from the GLM for each window will be referred to as “beta”, and the coefficients from the linear models will be referred to using the Greek letter $\upbeta$. Time was treated as a discrete variable with integer values of 1 through 3 (for the first through the third window) and was mean centered, and condition was coded as a binary variable with one of the conditions placed in the intercept. The condition in the intercept was the Neutral condition in the cue-reactivity LME, the Go condition in the response inhibition LME, and response inhibition during neutral cue exposure was in the methamphetamine-related response inhibition LME. This means that ${\upbeta }_{2}$ is a straightforward estimate of the contrast between the beta values associated with the two conditions in the model, and ${\upbeta }_{3}$ is the coefficient of the interaction of this condition with time. A random intercept was included to account for subject-level variations in the beta coefficients associated with the conditions in each model.

To guard against false positives and given the number of tests performed, regions with significant main effects of time and condition and those with a significant condition-by-time interaction effect were extracted after a False Discovery Rate (FDR) correction with a threshold of *p* < 0.001. Since a significant interaction term in an LME model indicates an intersection of the lines associated with different levels of one variable drawn across the levels of the other variable, observing a significant interaction of condition and time variables in a brain region in the LME models would mean two things: firstly, that the neural activity associated with one or both conditions is temporally evolving across the three windows (since two flat activation lines would not intersect) and secondly, that the evolution of neural activity differs between the two conditions in the model. Accordingly, it could be said that regions with a significant condition-by-time interaction in the first LME show “temporally dynamic cue-reactivity”, regions with a significant condition-by-time interaction in the second LME show “temporally dynamic response inhibition, and regions with a significant condition-by-time interaction in the third LME show “temporally dynamic methamphetamine-related response inhibition”. For each model, the temporal evolution of activity associated with either condition was examined by plotting the beta values (those from the GLM models in each window) for the relevant conditions across the three windows.

### Correlation of dynamic activity with behavioural and clinical data

After the identification of regions with dynamic activity in the group-level analysis, the relationship between the strength of this dynamic activity for each pair of conditions and behavioral and clinical data was investigated. For every ROI exhibiting a significant condition-by-time interaction in each model, separate linear models were fit for each subject with the form:

2 $${\varvec{\upbeta}}={\beta }_{0}+{\beta }_{1} \times condition+{\beta }_{2} \times time+{\beta }_{3} \times condition{:} \; time+ \epsilon,$$

where ${\varvec{\upbeta}}$ is a merged vector of the 6 beta coefficients associated with either of a pair of conditions across the three windows for the participant, and is estimated as a linear combination of time, condition, and their interaction. The estimated ${\beta }_{3}$ coefficients from these subject-level models, the slope of the interaction term, reflects the neural activity associated with one condition in the model contrasted to the other condition (which is placed in the intercept). Thus, in the subject-level cue-reactivity linear models (estimated in ROIs where the cue-reactivity LME indicates dynamic activity on the group level) the slope would represent sensitization to drug versus neutral cues across time, in the inhibition linear models it would represent escalating activation when inhibiting versus not inhibiting pre-potent responses (on NoGo and Go trials, respectively), and in the methamphetamine-related inhibition models it would be a subject-level reflection of an increased neural load required to successfully inhibit responses in the presence of drug versus neutral stimuli.

For each dynamically activated region in one of the three LMEs, individuals with positive $\upbeta$ values on subject-level linear models were compared to those with negative $\upbeta$ values in terms of the following variables using t tests: drug use severity (in terms of grams of methamphetamine used and the amount of money spent procuring drugs in the previous month), age of onset and duration of drug use, total number of risky behaviours, impulsivity (based on the BIS), Go/NoGo task performance indices (omission and commission error rate and reaction time), and craving before and after scanning (based on the VAS). The correlations of subject ${\beta }_{3}$ values and these variables were also explored (with and without dichotomization) and the results are presented in the supplementary materials. Given the large number of comparisons, FDR-corrected thresholds of *p* < 0.05 were used for these tests.

## Results

### Dynamic cue-reactivity

In the cue-reactivity LME, the main effect of condition (*DSNG* + *DSG* vs. *NSNG* + *NSG*) was significant in the medial superior frontal gyrus, sensory thalamus, paracentral lobule, superior temporal gyrus, rostroventral inferior parietal lobule, post-central gyrus, hypergranular insula, right caudodorsal cingulate gyrus, medio-ventral occipital cortex, and left sensory thalamus (Supplementary Fig. S1). The main effect of time (across three windows) was significant in the right lateral superior temporal gyrus (t = − 5.42, *p-*value = 3.0e−05) and bilateral caudal parahippocampal gyri (left (t = − 5.1, *p-*value = 2.0e−04), right (t = − 5.6, *p-*value = 2.0e−05)), right rostroposterior superior temporal sulcus (t = − 4.7, *p-*value = 0.001) and bilateral medial precunei (left (t = − 5.4, *p-*value = 3.0e−05), right (t = − 4.9, *p-*value = 5.0e−04)) (Supplementary Fig. S1).

**Figure 2:**
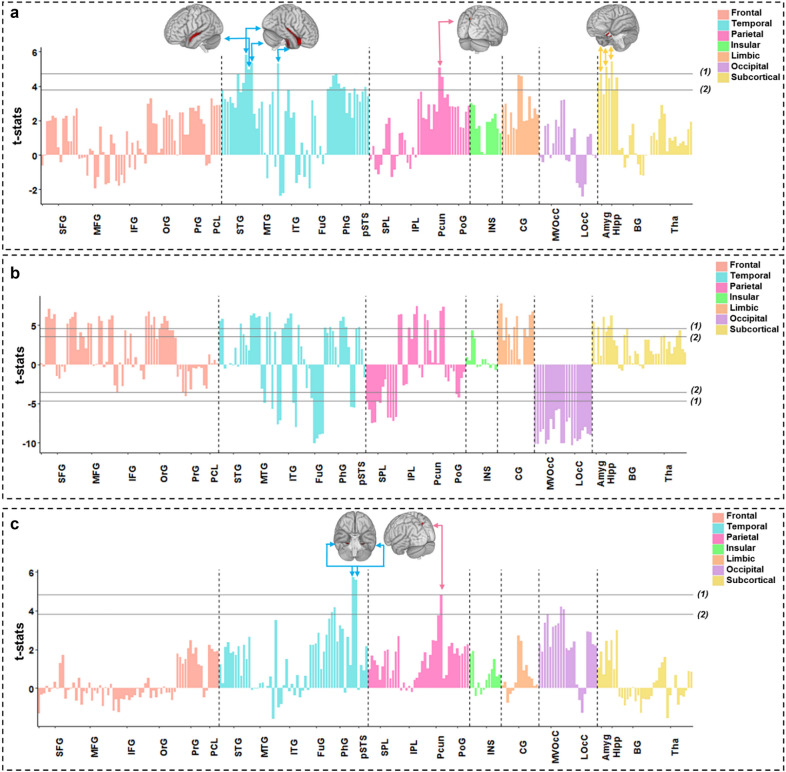
Condition by time interactions in the three linear mixed effects (LME) models. Bars show the coefficient of the condition by time (three windows) interaction term in the three LMEs (with condition: time as a fixed effect and subjects as a random effect) for each Brainnetome (BNA) subregion. 3D brains are those with significant activations in whole brain analyses. (**a**) Coefficient of interaction in the cue-reactivity LME ((Drug Successful NoGo + Drug Successful Go) or (Neutral Successful NoGo + Neutral Successful Go)). (**b**) Coefficient of interaction in the response inhibition LME ((Drug Successful NoGo + Neutral Successful NoGo) or (Drug Successful Go + Neutral Successful Go)). (**c**) Coefficient of interaction in the methamphetamine-related response inhibition LME ((Drug Successful NoGo-Drug Successful Go) or (Neutral Successful NoGo-Neutral Successful Go)). (1) FDR corrected *p*-value < 0.001. (2) FDR corrected *p*-value < 0.05. LMEs were implemented and visualized using R software version 3.6.2 (https://www.r-project.org/). 3D brains were visualized using FSLeyes, the FSL image viewer (https://open.win.ox.ac.uk/pages/fsl/fsleyes/fsleyes/userdoc/install.html). *SFG* superior frontal gyrus, *MFG* middle frontal gyrus, *IFG* inferior frontal gyrus, *OrG* orbital gyrus, *PrG* precentral gyrus, *PCL* paracentral lobule, *STG* superior temporal Gyrus, *MTG* middle temporal gyrus, *ITG* inferior temporal gyrus, *FuG* fusiform gyrus, *PhG* parahippocampal gyrus, *pSTS* posterior superior temporal sulcus, *SPL* superior parietal lobule, *IPL* inferior parietal lobule, *Pcun* precuneus, *PoG* postcentral gyrus, *INS* insular gyrus, *CG* cingulate gyrus, *MVOcC* medioventral occipital cortex, *LOcC* lateral occipital cortex, *Amyg* amygdala, *Hipp* hippocampus, *BG* basal ganglia, *Tha* thalamus.

The condition-by-time interaction (*condition: time*) in the cue-reactivity LME is significant in the right lateral superior temporal gyrus (t = 5.79, *p-*value = 5.0e−06), bilateral rostral superior temporal gyri (left: t = 4.92, *p-*value = 4.0e−04, right: t = 5.3, *p-*value = 6.0e−05) and right inferior temporal gyrus (t = 5.28, *p-*value = 6.0e−05), the left precuneus (t = 5.04, *p-*value = 2.0e−04), the right medial (t = 4.69, *p-*value = 0.001) and lateral amygdala (t = 5.09, *p-*value = 2.0e−04), and the right rostral hippocampus (t = 5.38, *p-*value = 4.0e−05) (Fig. 2a). Among these ROIs, only the right rostral superior temporal gyrus shows a significant condition effect (t = 4.16, *p-*value = 0.007) but all except left rostral superior temporal gyrus and right medial amygdala show a significant negative main effects of time (Table 2).

**Table 2:** Regions with significant condition by time interactions in the cue-reactivity and methamphetamine-related response inhibition linear mixed effects (LME) models. All *p-*values are FDR-corrected. FDR p-value threshold: 0.001. The p-values less than 1.0e−8 are considered as zero. The β, SE, and t-value are rounded to two decimal places. The response inhibition LME model revealed 107 regions with significant condition-by-time interactions, and complete results are shown in the Supplementary Table S1. In this table, results are shown only for regions implicated in a recent meta-analysis of fMRI studies with Go-NoGo tasks. FDR p-value threshold: 0.05. *β* Beta value, *SE* Standard Error, *STG* superior temporal gyrus, *ITG* inferior temporal gyrus, *Pcun (dmPOS)* dorsomedial parietooccipital sulcus part of precuneus, *Amyg* amygdala, *Hipp* hippocampus, *PhG* parahippocampal gyrus, *MFG* middle frontal gyrus, *RIFG (opercular)* right opercular part of inferior frontal gyrus, *PrG* precentral gyrus, *RCG* right cingulate gyrus, *LSPG* left superior parietal gyrus, *RMTG* right middle temporal gyrus, *RAngular* right angular gyrus, *LInsula* left insula.

Cue-reactivity (Drug Successful NoGo + Drug Successful Go) > (Neutral Successful NoGo + Neutral Successful Go)												
Location in BNA	Condition				Time				Condition: time			
	β	SE	t-value	*p*-value	β	SE	t-value	*p*-value	β	SE	t-value	*p*-value
Right STG (rostral)	0.08	0.02	4.16	0.0078	− 0.06	0.01	− 3.81	0.0378	0.13	0.02	5.30	0.00006
Left STG (rostral)	0.03	0.01	1.92	1	− 0.04	0.01	− 2.81	1	0.11	0.02	4.92	0.0004
Right STG (lateral)	0.04	0.01	2.23	1	− 0.09	0.02	− 5.42	0.00003	0.14	0.02	5.79	0.000005
Right ITG (intermediate ventral)	0.03	0.03	0.90	1	− 0.13	0.03	− 4.4	0.0031	0.21	0.04	5.28	0.00006
Left Pcun (dmPOS)	0.01	0.03	0.44	1	− 0.10	0.02	− 4.62	0.0013	0.16	0.03	5.04	0.0002
Right Amyg (medial)	0.03	0.02	1.24	1	− 0.04	0.02	− 2.44	1	0.12	0.03	4.69	0.00010
Right Amyg (lateral)	0.03	0.02	1.53	1	− 0.10	0.02	− 4.48	0.0027	0.16	0.03	5.09	0.0002
Right Hipp (rostral)	0	0.02	0.04	1	− 0.07	0.02	− 3.83	0.03514	0.15	0.03	5.38	0.00004

Response inhibition (Drug Successful NoGo + Neutral Successful NoGo) > (Drug Successful Go + Neutral Successful Go)												
Location in BNA	Condition				Time				Condition: time			
	β	SE	t-value	*p*-value	β	SE	t-value	*p*-value	β	SE	t-value	*p*-value
Right MFG	− 0.104	0.02	− 6.55	0	0.06	0.02	0.03	0.003	0.12	0.03	4.08	0.00006
Left MFG	− 0.1	0.02	− 3.82	0.0002	− 0.043	0.02	− 1.91	0.05	0.17	0.03	5.31	0.0000002
Right IFG	0.12	0.03	4.24	0.00003	0.04	0.02	1.62	0.0161	− 0.13	0.04	− 3.68	0.00028
Right PrG	0.06	0.03	1.92	0.05508	0.05	0.03	1.86	0.0627	− 0.15	0.04	− 4.07	0.00006
Left PrG	0.06	0.03	1.85	0.06505	0.05	0.03	1.76	0.0799	− 0.14	0.04	− 3.58	0.00041
Right CG	− 0.22	0.03	− 6.57	0	− 0.1	0.03	3.29	0.0013	0.27	0.04	6.35	0
Right Angular	− 0.23	0.03	− 8.05	0	− 0.1	0.03	− 4.09	0.00005	0.26	0.03	7.39	0
Left Supermarginal	− 0.09	0.02	− 3.98	0.000089	− 0.04	0.02	− 1.86	0.0638	0.13	0.03	4.69	0.000004
Left SPG	0.13	0.03	4.61	0.0021	0.06	0.02	2.39	0.0745	− 0.26	0.03	− 5.97	0.00002
Right MTG	− 0.2	0.03	− 6.48	0.00000005	− 0.08	0.03	− 3.25	0.0031	0.24	0.03	6.54	0
Left Insula	− 0.13	0.03	− 4.16	0.000044	− 0.06	0.03	− 2.41	0.0167	0.16	0.04	4.3	0.000024

Methamphetamine-related response inhibition (Drug Successful NoGo −Drug Successful Go) > (Neutral Successful NoGo − Neutral Successful Go)												
Location in BNA	Condition				Time				Condition: time			
	β	SE	t-value	*p*-value	β	SE	t-value	*p*-value	β	SE	t-value	*p*-value
Right Pcun (dmPOS)	− 0.06	0.02	− 2.51	0.06	− 0.06	0.02	− 2.79	0.78	0.14	0.02	4.83	0.0005
Right PhG (medial)	− 0.19	0.03	− 3.91	0.014	0.002	0.02	0.08	1	0.19	0.03	5.59	0.00001
Left PhG (medial)	− 0.11	0.02	− 4.00	0.011	0.003	0.02	0.125	1	0.20	0.03	5.75	0.000006

**Figure 3:**
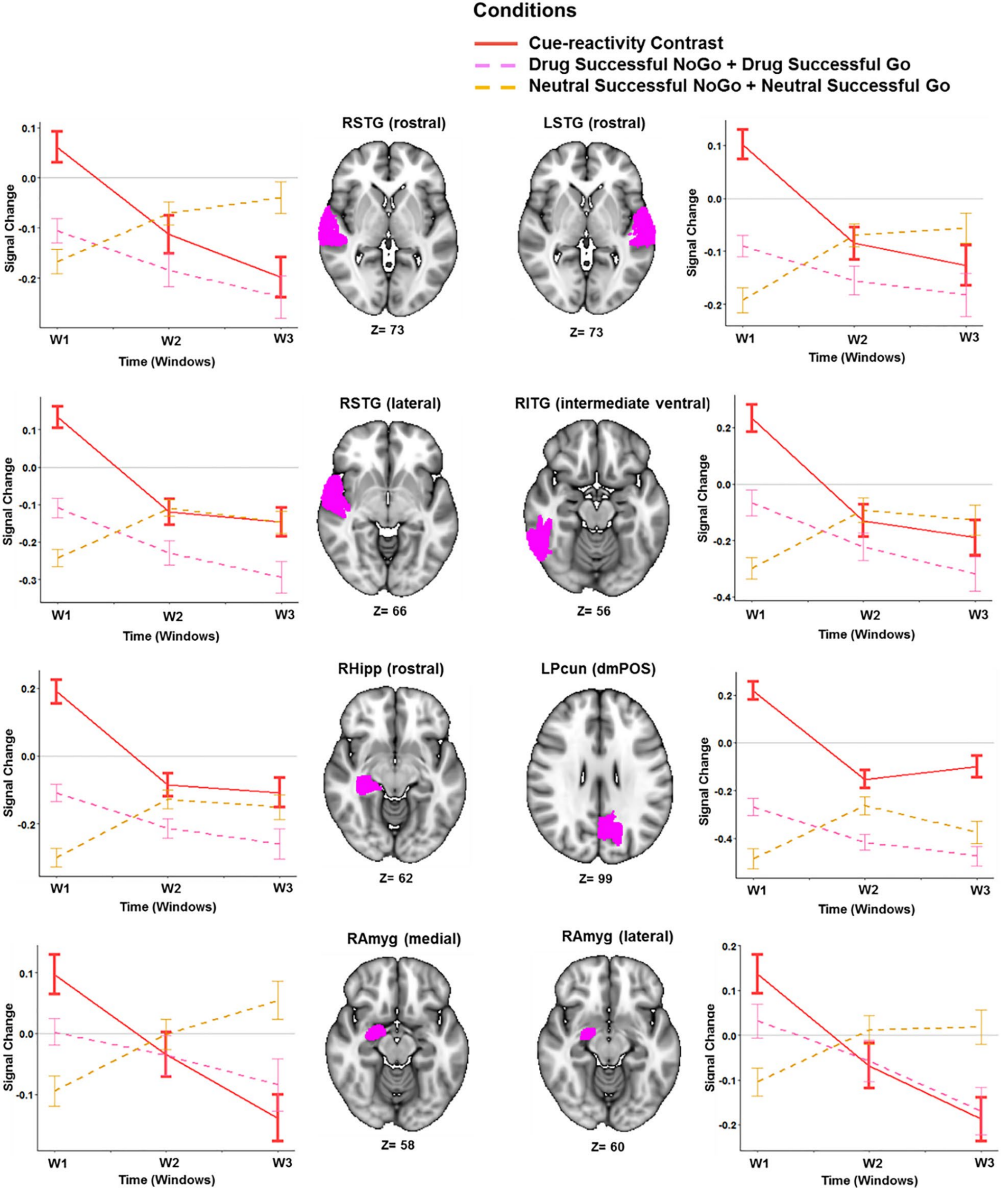
Temporal behavior of ROIs with significant condition by time interactions in the cue-reactivity linear mixed effects (LME) model. Lines show the average main effect of Drug (Drug Successful NoGo + Drug Successful Go) or Neutral (Neutral Successful NoGo + Neutral Successful Go) cue-exposure condition in the Brainnetome (BNA) regions or the result of Drug vs. Neutral contrast, and error bars show the standard error of z-statistic values across 53 methamphetamine use disorders (MUD) at each temporal window (FDR corrected *p-*value < 0.001). The graphs were implemented and visualized using R software version 3.6.2 (https://www.r-project.org/). 3D brains were visualized using FSLeyes, the FSL image viewer (https://open.win.ox.ac.uk/pages/fsl/fsleyes/fsleyes/userdoc/install.html). *RSTG* right superior temporal gyrus, *LSTG* left superior temporal gyrus, *RITG* right inferior temporal gyrus, *RHipp* right hippocampus, *LPcun* left precuneus, *RAmyg* right amygdala.

Subcortical regions such as the amygdala and hippocampus, as well as the superior temporal gyrus and precuneus show a decreasing response over time (habituation) to drug cues compared to neutral cues (Fig. 3).

### Dynamic response inhibition

The response inhibition LME model revealed many regions with a significant effect of condition *(DSNG* + *NSNG* vs*. DSG* + *NSG)* (Supplementary Fig. S1) and significant condition-by-time interactions (Fig. 2b). The main effect of time was significant in only a handful of regions however, including the bilateral fusiform gyri (left (t = 5.53, *p-*value = 2.0e−05), right (t = 5.2, *p-*value = 1.0e−04)), right medial precuneus (t = − 4.93, *p-*value = 3.0e−04), medio-ventral and lateral occipital cortices (corrected *p*-value < 0.001) (Supplementary Fig. S2), (Supplementary Table S1).

**Figure 4:**
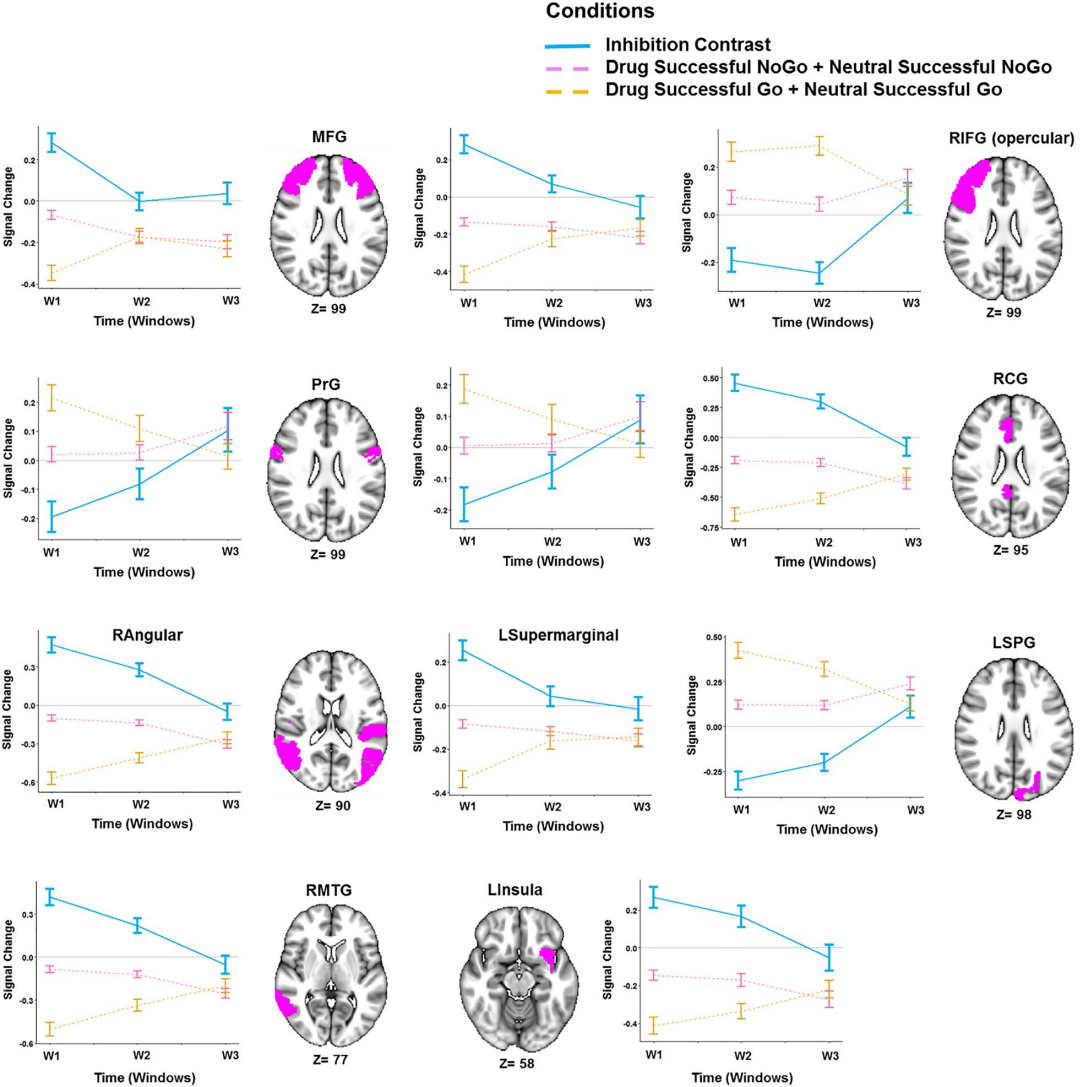
Temporal behavior of ROIs with significant condition by time interactions in the response inhibition linear mixed effects (LME) model. Lines show the average main effect of NoGo (Drug Successful NoGo + Neutral Successful NoGo) or Go (Drug Successful Go + Neutral Successful Go) inhibition condition in the Brainnetome (BNA) regions or the result of Go vs. NoGo contrast, and error bars show the standard error of z-statistic values across 53 methamphetamine use disorders (MUD) at each temporal window (FDR corrected *p-*value < 0.001). The graphs were implemented and visualized using R software version 3.6.2 (https://www.r-project.org/). 3D brains were visualized using FSLeyes, the FSL image viewer (https://open.win.ox.ac.uk/pages/fsl/fsleyes/fsleyes/userdoc/install.html). *MFG* middle frontal gyrus, *RIFG (opercular)* right opercular part of inferior frontal gyrus, *PrG* precentral gyrus, *RCG* right cingulate gyrus, *LSPG* left superior parietal gyrus, *RMTG* right middle temporal gyrus, *RAngular* right angular gyrus, *LInsula* left insula.

Since dynamic inhibition was observed in 107 regions, we only visually explored the temporal inhibitory and non-inhibitory behavior of regions shown to be involved in response inhibition in a recent meta-analysis of fMRI Go-NoGo tasks^53^. The bilateral middle frontal gyri (left (t = 5.3, *p-*value = 2.0e−07), right (t = 4.08, *p-*value = 6.0e−05)), right cingulate gyrus (t = 6.3, *p-*value = 6.0e−10), left insula (t = 4.3, *p-*value = 2.4e−05), right angular gyrus (t = 7.3, *p-*value = 4.0e−10), right middle temporal gyrus (t = 6.54, *p-*value = 7.0e−08) and left supramarginal gyrus (t = 4.69, *p-*value = 4.0e−6) showed falling inhibitory activations (habituation) to NoGo cues, while the bilateral precentral gyri (left (t = − 3.58, *p-*value = 4.1e−04), right (t = − 4.07, *p-*value = 6.0e−05)), right superior parietal lobule (t = − 5.97, *p-*value = 2.0e−05), and right opercular inferior frontal gyrus (t = − 3.68, *p-*value = 2.8e−04)) showed increasing contrasts (sensitization) (Fig. 4).

### Dynamic methamphetamine-related response inhibition

In the LME modeling response inhibition during exposure to methamphetamine vs neutral cues, the main effect of condition *(DSNG* − *DSG* vs. *NSNG* − *NSG)* is highly significant across much of the frontal cortex, superior temporal gyrus, inferior parietal lobule, precuneus, post-central gyrus, dorsal insula, medioventral occipital cortex, and basal ganglia (corrected *p*-value < 0.001) (Supplementary Fig. S1). Regions with a significant main effect of time are the inferior temporal gyrus, fusiform gyrus, parahippocampal gyrus , precuneus, right dorsal and ventral cingulate gyrus, lateral occipital cortex, and hippocampus (corrected p-value < 0.001) (Supplementary Fig. S2).

**Figure 5:**
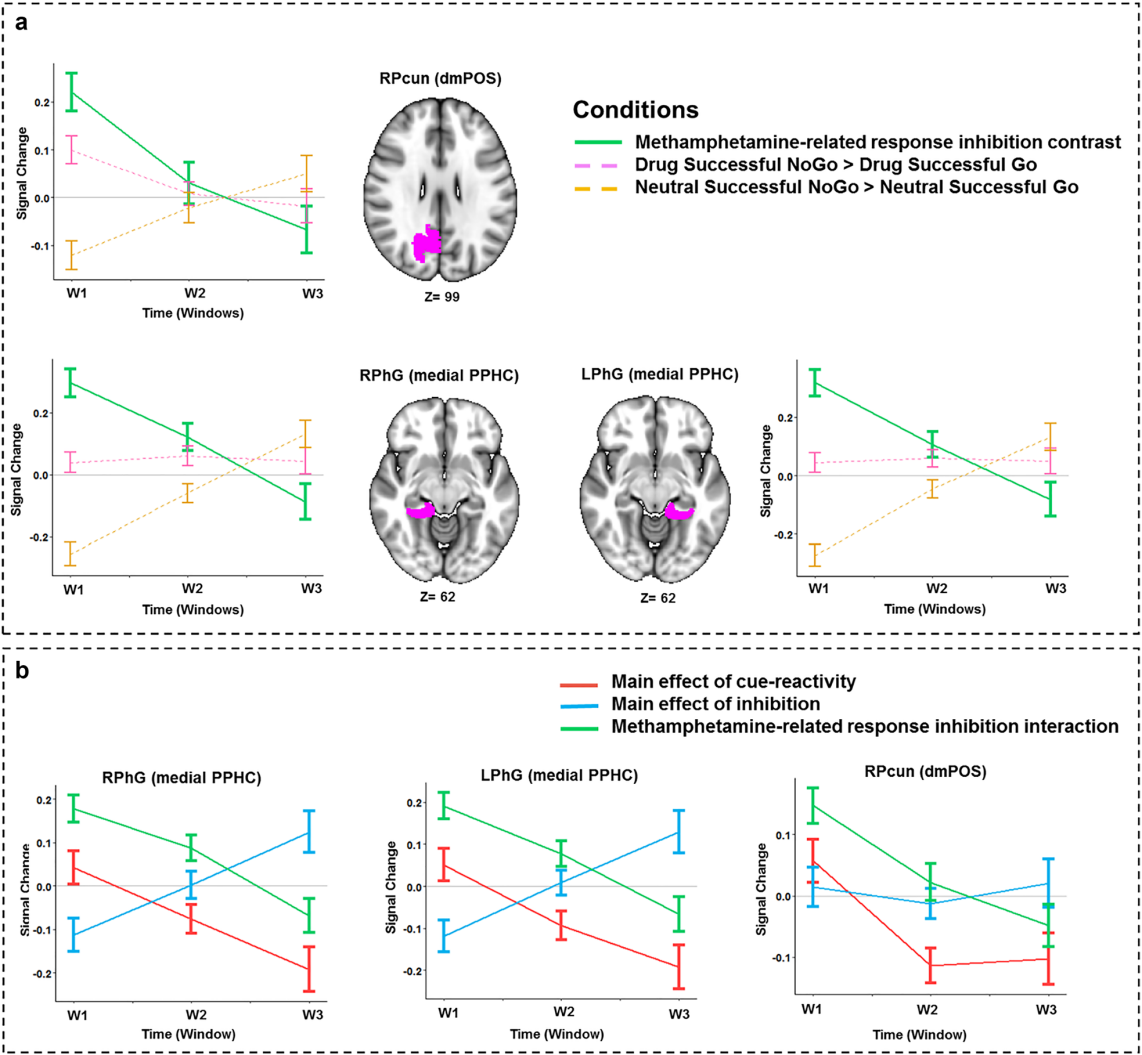
(**a**) Temporal behavior of ROIs with significant condition by time interactions in the methamphetamine-related response inhibition linear mixed effects (LME) model. Lines show the average main effect of Drug-related inhibition (Drug Successful NoGo-Drug Successful Go) or Neutral-related inhibition (Neutral Successful NoGo-Neutral Successful Go) condition or the result of methamphetamine-related response inhibition contrast in the Brainnetome (BNA) regions, and error bars show the standard error of z-statistic values across 53 methamphetamine use disorders (MUD) at each temporal window (FDR corrected *p-*value < 0.001). The graphs were implemented and visualized using R software version 3.6.2 (https://www.r-project.org/). 3D brains were visualized using FSLeyes, the FSL image viewer (https://open.win.ox.ac.uk/pages/fsl/fsleyes/fsleyes/userdoc/install.html). (**b**) Temporal behavior of the main effects of cue-reactivity, response inhibition and methamphetamine-related response inhibition contrasts in the ROIs with significant condition by time interactions extracted from methamphetamine-related response inhibition LME model (FDR corrected *p*-value < 0.001). The lines show mean parameter estimates, and the error bars show the standard error of z-statistic values across 53 methamphetamine use disorders (MUD). *RPcun (dmPOS)* dorsomedial parietooccipital sulcus part of right precuneus, *RPhG* right parahippocampal gyrus, *LPhG* left parahippocampal gyrus.

The right precuneus shows a significant condition-by-time interaction (t = 4.83, *p*-value = 5.6e−04) with an attenuating inhibitory response while viewing drug-related cues *(DSNG − DSG)* but not during neutral cue exposure *(NSNG − NSG).* The main effects of condition and time are not significant in this region individually. Bilateral medial parahippocampal gyri also show significant condition-by-time interactions (right: (t = 5.59, *p*-value = 1.40e−05), left: (t = 5.75, *p*-value = 6.0e−06)) (Fig. 2c), and have temporally stable activations during response inhibition in the presence of drug-related cues but escalating activations during neutral cue exposure (Fig. 5a). The main effect of condition was significant in these regions (right: (t = − 3.9, *p*-value = 0.014), left: (t = − 4.0, *p*-value = 0.011)), unlike the main effect of time (Table 2).

For the three ROIs with a dynamic involvement in methamphetamine-related response inhibition, values of three contrasts for cue-reactivity, response inhibition and methamphetamine-related response inhibition were estimated across the overlapping windows for further exploration, and the results are illustrated in Fig. 5b. The parahippocampal gyri show a habituation to drug vs. neutral cues *(DSNG* + *DSG* > *NSNG* + *NSG)* and to methamphetamine-related response inhibition *(DSNG − DSG* > *NSNG − NSG)*, but increasing values of the response inhibition contrast *(DSNG* + *NSNG* > *DSG* + *NSG)* across time. In the right precuneus as well, the cue-reactivity and methamphetamine-related response inhibition contrasts decrease in value over time but the response inhibition contrast remains mostly stable.

### Correlates of dynamic brain activity

Several significant associations were observed in the exploratory investigations of potential links between behavioral and clinical variables and participant activation slopes in dynamically activated regions. Individuals with positive cue-reactivity activation slopes in the left superior temporal gyrus and the right hippocampus had lower omission error rates on the Go-NoGo task (*p*-values = 0.017 and 0.028, respectively) and shorter average reaction times (*p*-values = 0.011 and 0.036, respectively) compared to those with negative activation slopes. Having a positive cue-reactivity slope in the right superior temporal gyrus was associated with greater methamphetamine use (*p*-value = 0.0009) and higher baseline craving (*p*-value = 0.013), but shorter average reaction times on the task (*p*-value = 0.013). Having a positive cue-reactivity slope in the left precuneus was associated with the amount of money spent procuring methamphetamine (*p*-value = 0.018) but lower motor impulsivity (p-value=0.022), and a positive slope in the right amygdala was associated with fewer risky behaviours (*p*-value = 0.039). Considering the response inhibition linear models, Individuals with positive activation slopes in the right superior temporal gyrus reported higher motor (*p*-value = 0.005) and total impulsivity (*p*-value = 0.001) and more money spent procuring methamphetamine (*p*-value = 0.016), but a lower craving after the task (*p*-value = 0.010). Having a positive response inhibition slope was associated with a longer average reaction time in the left parahippocampus (*p*-value = 0.041), but a shorter average reaction time in the right parahippocampus (*p*-value = 0.013) (Table 3).

**Table 3:** Results of t-tests between groups with positive interaction β values from linear model, and groups with negative β values in the cue reactivity and response inhibition contrasts. There were no significant regions in the methamphetamine-related response inhibition contrast.

Brainnetome ROI	Behavioral variable	t-value	P-value	
			Uncorrected	FDR corrected
**Cue reactivity**				
Left lateral superior temporal gyrus	Omission error	− 2.66	0.017	0.113
	Total reaction time	− 2.76	0.011	0.048
Right lateral superior temporal gyrus	Pre VAS	2.61	0.013	0.104
	Total reaction time	− 2.74	0.013	0.049
Right rostral superior temporal gyrus	Dosage	3.59	0.0009	0.007
Left precuneus	Barrat motor	− 2.54	0.022	0.176
	Total drug cost in the last month	2.47	0.018	0.145
Right lateral amygdala	Total risky behavior	− 2.27	0.039	0.195
Right rostral hippocampus	Omission error	− 2.390	0.028	0.114
	Total reaction time	− 2.204	0.036	0.098

Brainnetome ROI	Behavioral variable	t-value	P-value	
			Uncorrected	FDR corrected
Response inhibition				
Right lateral superior temporal gyrus	Barrat sum	3.57	0.001	0.006
	Barrat motor	2.97	0.005	0.028
	Total drug cost in the last month	2.53	0.016	0.082
	Post vas	− 2.64	0.010	0.048
Left parahippocampal gyrus	Total reaction time	2.17	0.041	0.103
Right parahippocampal gyrus	Total reaction time	− 2.67	0.013	0.069

Besides the t tests, linear relationships between the value of regional dynamic activation slopes and behavioral or clinical variables were also explored. The results of these comparisons are presented in the supplementary materials (Supplementary Tables S2, S3, S4). For cue reactivity activation slopes, omission error is negatively correlated with interaction β coefficients in the superior temporal gyri, left precuneus, right medial amygdala, and right rostral hippocampus after FDR correction. For the response inhibition contrast, amount of money spent procuring methamphetamine is correlated with the interaction β coefficient in the right lateral superior temporal gyrus after FDR correction. There were no significant correlations between methamphetamine-related response inhibition slopes and clinical and behavioral data.

## Discussion

This exploratory study is an investigation of temporally dynamic regional brain activation patterns underlying cue-reactivity, response inhibition, and methamphetamine-related response inhibition in individuals with MUD. Sliding window techniques are relatively common in dynamic functional connectivity analyses, and in general rest on estimating the parameters of a model for overlapping windows in time before comparing estimates across windows^55,56^. Despite decades of accumulated evidence for temporal variation in regional sensitization and habituation in cognitive neuroscience^31–33^, dynamic analyses of regional activation in addiction remain rare and this is the first study exploring dynamic response inhibition in the context of methamphetamine cue-reactivity. This was done by fitting linear mixed effects models to model the interaction of time with a cue-reactivity contrast, a response inhibition contrast, and a methamphetamine-related response inhibition contrast, after estimating regressor coefficients in group-level models for each window. In essence, this study extends the dynamic analysis approach proposed and replicated by Ekhtiari et al.^37^ for the dynamic analysis of cue-reactivity in methamphetamine and opioid using individuals to investigate dynamic methamphetamine-related response inhibition, which has been investigated in a few studies using conventional analyses with largely similar contrasts^28,57–59^.

### Dynamic cue-reactivity

Dynamic cue-reactivity was observed in the bilateral superior temporal gyrus, the right amygdala, and rostral hippocampus, and the left precuneus and inferior temporal gyrus. Many of these regions have previously been indicated in methamphetamine cue-reactivity^16,17,19^, and drug cue-reactivity more widely^60,61^. Notably, dynamic amygdala activity with a similar downward slope over time has been observed in two recent cue-reactivity studies in individuals with MUD and opioid use disorder^37,38^. A study on individuals with heroin use disorder estimating dynamic causal modeling parameters in overlapping windows has also demonstrated craving inputs to the amygdala increase during a cue-reactivity task, and that the dorsolateral prefrontal cortex’s modulatory impact on the connection between the ventromedial prefrontal cortex and the amygdala decreases over time^62^. Ekhtiari et al. also similarly reported bilateral dynamic cue-reactivities in the superior temporal gyrus, but they observed an initially escalating and subsequently decreasing activation whereas we observed a consistent habituation response^37^. Broadly, our results suggest generalized habituation to drug cues across the task duration. The dynamic cue-reactivity LME showed no significant condition-by-time interactions in the ventromedial prefrontal cortex and the ventral striatum, indicating a lack of dynamic activity, unlike another dynamic study using similar analytical procedures^37^. Unexpectedly, these regions also showed no static activity, potentially showing that they were not recruited by our task components.

As expected, a positive cue-reactivity slope across right rostral and lateral temporal regions and the left precuneus were associated with higher baseline craving and drug use amount, indicating that an escalating neural response to drug cues is associated with more intense craving and real-life drug use. Another strikingly consistent but unexpected observation however, is that this sensitization was associated with better response inhibition: participants with positive cue-reactivity slopes in the right hippocampus and bilateral superior temporal gyri reported less impulsivity and also showed better response-inhibitory task performance, and similarly, those with left precuneal and right amygdalar sensitization reported lower motor impulsivity and fewer risky behaviors. A possible explanation for the association between escalating cue-reactivity and improved response inhibition might be that a greater engagement with drug cues facilitates attention to the response inhibitory task. Indeed, improvements in response inhibition during cue-reactivity have been reported in individuals with problem gambling before^59^. In regression analyses without dichotomization also, omission error is negatively correlated with interaction β coefficients in the superior temporal gyri, left precuneus, right medial amygdala, and right rostral hippocampus. This further supports the hypothesis that the escalating engagement with drug versus neutral cues in these regions may contribute to better performance on the task.

### Dynamic response inhibition

More than a hundred regions in our LME model showed dynamic response inhibitory activity. This may not be surprising, as response inhibition is associated with large-scale neural activity^54^ and dynamic brain network reconfiguration^41^. Also, notable is that dynamic prefrontal activations were also observed in the response inhibition model, whereas only FDR-uncorrected prefrontal activations were observed in the other two models (cue-reactivity and methamphetamine-related response inhibition). There have been reports of prefrontal sensitization to salient cues^36^, and it has been observed that the prefrontal cortex is implicated in the dysfunctional behavioral regulation seen in the MUD during response control tasks^22^. The observation of dynamic activity in prefrontal regions was expected, given their involvement in inhibitory control networks^51^ and response inhibition in substance use disorders^63,64^.

Most of the regions involved in response inhibition in a recent meta-analysis of Go-NoGo tasks^54^ had dynamic activation patterns in this study. Notably, while dynamic cue-reactivity was associated with a generalized habituation effect, these regions showed two broad temporal activation patterns. The middle temporal gyrus, the left insula, the right cingulate gyrus, the right middle temporal gyrus, and supramarginal gyrus showed falling inhibitory activations while the precentral gyrus, left superior parietal lobule, and opercular frontal gyrus showed increasing activations (sensitization). This might reflect differences in response inhibitory processes that these regions contribute to, such as error monitoring and attentional control^65,66^, or the involvement of these regions in other networks that interact with the response inhibition network in individuals with substance use disorders, such as the insula in the salience network or the middle frontal gyrus in self-directed processing^14^. Compared to the cue-reactivity contrast, increasing response-inhibition-associated activity showed more mixed associations with behavioral variables in t-test comparisons with individuals who had decreasing activation across a number of regions. An increasing response-inhibition contrast in the right superior temporal gyrus was associated with both higher impulsivity and higher drug use but with a lower craving after the task, while parahippocampal sensitization to response inhibition was associated with both shorter and longer reaction times in the left and right parahippocampi, respectively. These contradictory associations may indicate that the dynamic neural correlates of response inhibition are reliable indicators of neither addiction severity nor impulsivity and inhibitory capacity, though they may also have resulted from unnoticed design flaws. Turning to regression analyses without dichotomization, amount of money spent procuring methamphetamine is correlated with the interaction β coefficient in the right lateral superior temporal gyrus after FDR correction.

### Dynamic methamphetamine-related response inhibition

The bilateral parahippocampal gyri and the right precuneus were the only regions with a dynamic methamphetamine-related response inhibition. Several meta-analyses have demonstrated that drug cue-reactivity is associated with heightened precuneal activation^67,68^, and based on the response inhibition literature, dopaminergic inhibition, and network decoupling of precuneal activity may be important for successful response inhibition^69–71^. Precuneal involvement in cue-reactivity in substance use disorders might be related to its role in the default mode network and self-referential processing in general^14^, and, interestingly, it has been argued that the precuneus might be an important node for the integration of contradictory executive control and cue-reactivity processes^72^. Considering the above, the decreasing activation associated with drug-related inhibition in the right precuneus may reflect a lessening effect of drug cues in hampering response inhibition across the task duration. Since it appears that the response inhibition contrast in the precuneus is mostly stable across time while cue-reactivity and methamphetamine-related response inhibition contrasts decline, habituation to drug cues or top-down suppression of precuneal cue-reactivity, rather than the role of the precuneus in response inhibition per se, maybe the responsible mechanisms.

The parahippocampus has also been implicated in substance use disorders. Addictive disorders are associated with parahippocampal gray matter changes^73^ and increases in its connectivity within the default mode network^74^, both the right and the left parahippocampus generally show higher activations in response to drug-related cues compared to neutral cues^67,75,76^, and response inhibition-associated parahippocampal dysfunction has been observed in individuals with substance use disorders compared to healthy controls^20^. As part of the default mode network and given its association with drug cue-reactivity, it was expected that similar to the precuneus, the cue-reactivity contrast in the parahippocampal gyri would decrease, reflecting both habituation processes and task-engagement-related suppression. Some evidence also exists for parahippocampal habituation during exposure to emotionally salient stimuli^77,78^ and for the role of the parahippocampus in the extinguishing of drug cue associations^79^. However, the parahippocampus is also involved in neural networks underlying associational memory and learning^80,81^ and might be activated to support learning during response inhibition tasks^14^. Indeed, increasing parahippocampal recruitment during a learning task has been observed before^82^. These dual roles of the parahippocampus in cue habituation and learning could explain why the cue-reactivity contrast decreased while the response inhibition contrast increased in the parahippocampal gyri during the task, and is supported by the observation that drug-related inhibition remained mostly stable, while inhibition during neutral cue exposure was associated with increasing parahippocampal activity.

An interesting observation in this study was the right-lateralization of dynamically active regions across the three contrasts. Some evidence exists that the right hemisphere may be more important in response inhibitory and attentional control processes^83,84^, and right lateralization of dynamic response to salient stimuli has been observed in the right amygdala, inferior parietal lobule, and hippocampus^31,85^. It has been argued that while the left amygdala is involved in sustained stimulus evaluation, the right amygdala might be more specialized for dynamic stimulus processing^34^. This may also explain why sensitization to response inhibition in the right parahippocampal gyrus was associated with better response inhibitory performance, but sensitization in the left parahippocampus had the opposite association.

In general, it appears that regions with temporally dynamic methamphetamine-related response inhibition increasingly engage in response-inhibitory processing while developing a habituation to drug cues. This may indicate a general pattern underlying repeated drug cue exposure, with neural resources gradually shifting to inhibit undesirable responses while a habituation to drug cues develops and the initial intensity of cue reactivity dissipates.

## Limitations

While the results of this exploratory investigation are promising, several limitations are important to point out. Firstly, we included no healthy control group, and so the specificity of observed patterns to individuals with MUD is unclear. Also, all participants were men, treatment seeking individuals MUD, limiting the generalizability of our observations. Regarding the task design, an inherent limitation introduced by our use of a mixed drug cue and negative emotional Go-NoGo task is the potential carry-over effects of salient cues on brain activity during subsequent blocks^86,87^. While such issues may be ameliorated by the choice of a blocked presentation of different cue types, the results are likely confounded by these effects. A further concern with our separation of the signal into three windows would be the resulting loss of power; and while we had a moderately sized sample, the reported observations need to be replicated in larger samples. Lastly, we used no measure of craving across the task duration, which would have allowed the analysis of temporal correlations between craving and neural activity, as in one recent study by Murphy et al.^38^.

## Conclusion

This study provides preliminary evidence that a mixed event-block Go-NoGo/cue-reactivity task can be used to assess the temporal dynamics of cue-reactivity, response inhibition, and methamphetamine-related response inhibition. The regions with a temporally dynamic response are involved in various neuro-cognitive aspects of addictive disorders. Notably, we observed dynamic amygdalar activity in both response inhibition and cue-reactivity contrasts, and there is extensive literature on the time-variance of amygdalar activity^31,34,37,88^, and it has been recently argued that amygdalar habituation is a more reliable index than mean amplitude^89^. Dynamic methamphetamine-related response inhibition was observed in regions in which the neural activations associated with cue-reactivity and response inhibition followed broadly opposing slopes across time, namely in the parahippocampal regions and the precuneus, suggesting that these regions may be important hubs where response inhibitory and cue-reactivity processes integrate. Investigating the dynamic interplays of cognitive processes in these regions may help biomarker development and suggest new targets for interventions, and it has been suggested that failures to inhibit precuneal cue-reactivity may predict relapse^90^, and impairments of parahippocampal habituation are associated with poorer treatment outcomes in cocaine users^39^. Future studies could make use of better power analyses, flexible sliding window sizes and inference methods, prospective designs, and replication across different populations and time-points to assess the stability, generalizability, and potential predictive utility of these dynamic activation parameters.

## Supplementary Information

Supplementary Information.

## Data Availability

The raw database for this study is available on reasonable request from the corresponding authors.

## Code availability

R scripts and condition beta values of this study are available on request from the corresponding authors.
