# Supplementary material for: Temporally dynamic neural correlates of drug cue reactivity, response inhibition, and methamphetamine-related response inhibition in people with methamphetamine use disorder
Source: Sci Rep. 2022 Mar 4;12:3567. doi: 10.1038/s41598-022-05619-8 (PMC8897423; doi:10.1038/s41598-022-05619-8)
Supplement: Supplementary file 1 — Supplementary Information. [file 41598_2022_5619_MOESM1_ESM.pdf]

Supplementary Materials

Temporally Dynamic Neural Correlates of Drug Cue Reactivity, Response Inhibition, and Methamphetamine-Related Response Inhibition in People with Methamphetamine Use Disorder

Sara Jafakesh[1], Arshiya Sangchooli[3], Ardalan Aarabi[4], Mohammad Sadegh Helfroush[1], Amirhossein Dakhili[6], Mohammad Ali Oghabian[7], Kamran Kazemi[1#], Hamed Ekhtiari[2#]

[1] Department of Electrical and Electronics Engineering, Shiraz University of Technology, Shiraz, Iran
[2] Laureate Institute for Brain Research (LIBR), Tulsa, OK, USA
[3] Iranian National Center for Addiction Studies (INCAS), Tehran University of Medical Science, Tehran, Iran.
[4] Faculty of Medicine, University of Picardie Jules Verne, Amiens, France
[5] Laboratory of Functional Neuroscience and Pathologies (LNFP), University Research Center (CURS), University Hospital, Amiens, France
[6] Medical Physics Department, Iran University of Medical Sciences, Tehran, Iran
[7] Medical Physics and Biomedical Engineering Department, Tehran University of Medical Sciences, Tehran Iran

Supplementary Figures

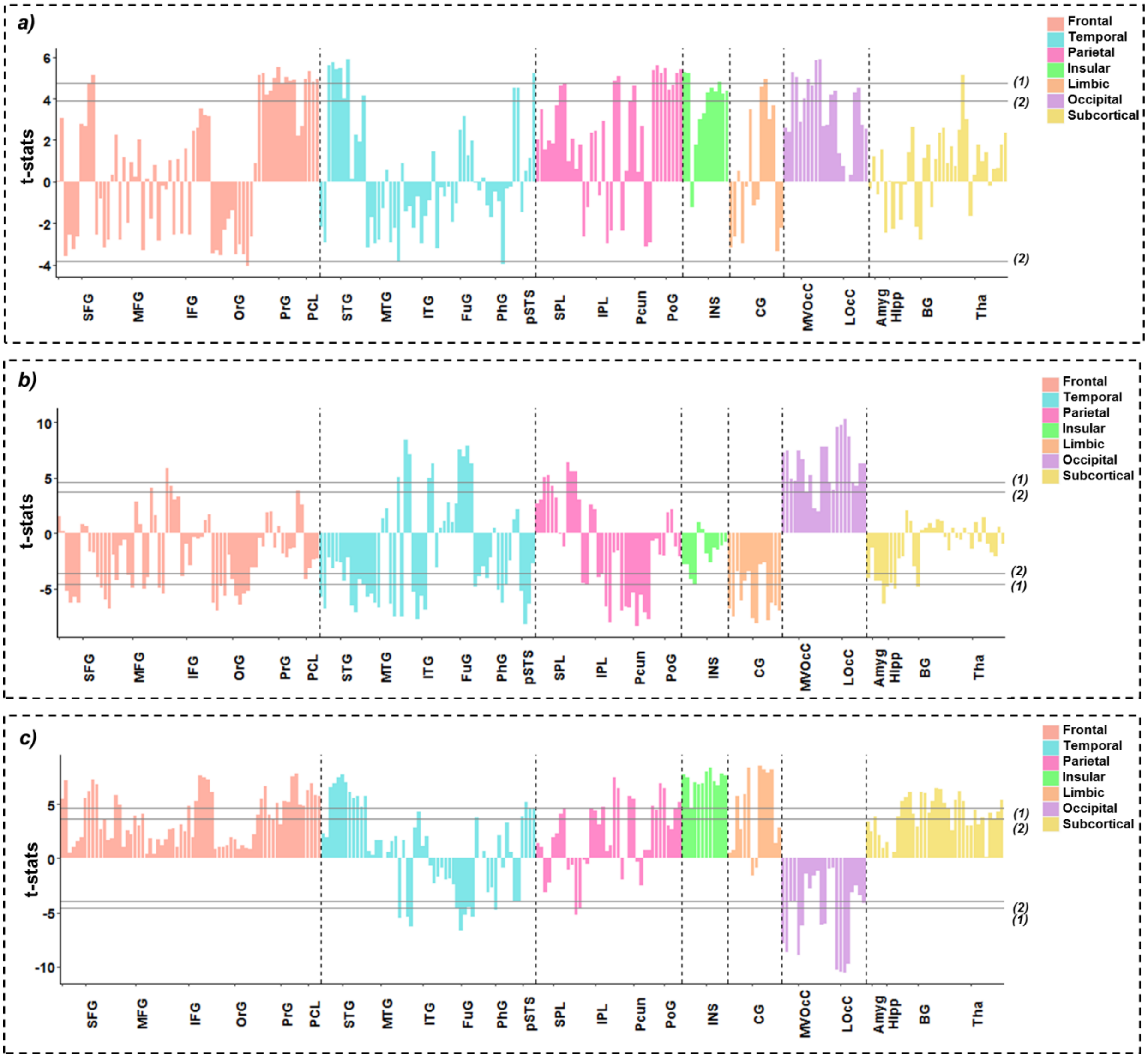

Supplementary Figure S1. The main effect of conditions in the three linear mixed effects (LME) models. Colored bars show the coefficient of the main effect of the condition term in the LME model for each Brainnetome (BNA) subregion**.** ***(a)*** The main effect of condition in the cue-reactivity LME ((Drug Successful NoGo+Drug Successful Go) or (Neutral Successful NoGo+Neutral Successful Go)). ***(b)*** The main effect of condition in the response inhibition LME ((Drug Successful NoGo+Neutral Successful NoGo) or (Drug Successful Go + Neutral Successful Go)). ***(c)*** The main effect of condition in the methamphetamine-related response inhibition contrast LME ((Drug Successful NoGo>Drug Successful Go) or (Neutral Successful NoGo>Neutral Successful Go)). (1) FDR corrected *p*-value<0.001. (2) FDR corrected *p*-value<0.05.

Linear Mixed Effect Models were analysed and visualized using R software version 3.6.2 (https://www.r-project.org/).

Abbreviation: SFG: superior frontal gyrus, MFG: middle frontal gyrus, IFG: inferior frontal gyrus, OrG: orbital gyrus, PrG: precentral gyrus, PCL: paracentral lobule, STG: superior temporal Gyrus, MTG: middle temporal gyrus, ITG: inferior temporal gyrus, FuG: fusiform gyrus, PhG: parahippocampal gyrus, pSTS:: posterior superior temporal sulcus, SPL: superior parietal lobule, IPL: inferior parietal lobule, Pcun: precuneus, PoG: postcentral gyrus, INS: insular gyrus, CG: cingulate gyrus, MVOcC: medioventral occipital cortex, LOcC: lateral occipital cortex, Amyg: amygdala, Hipp: hippocampus, BG: basal ganglia, Tha: thalamus.

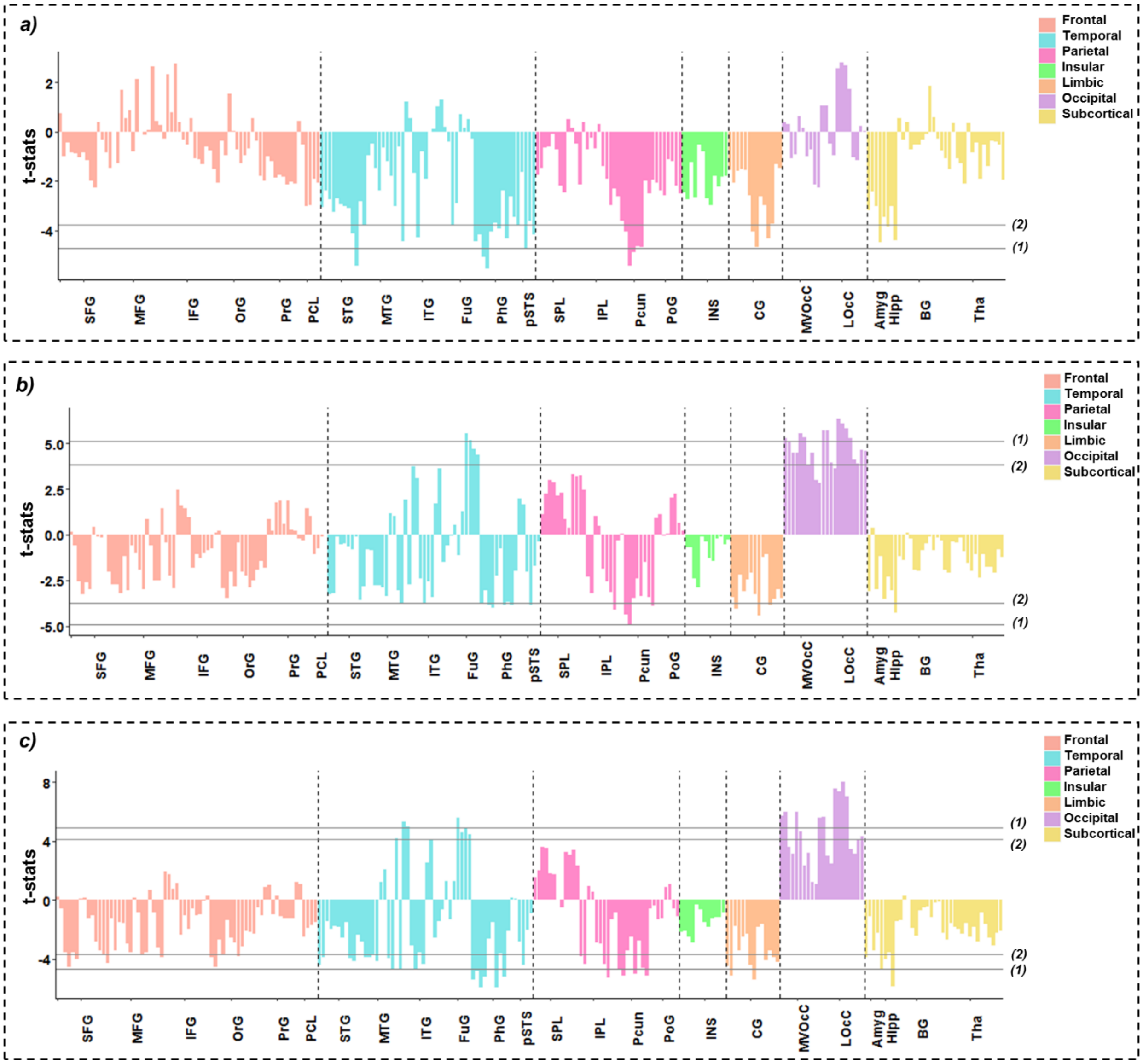

Supplementary Figure S2. The main effect of time (three windows) in the three linear mixed effects (LME) models. Colored bars show the coefficient of the main effect of the time term in the LME model for each Brainnetome (BNA) subregion**.** ***(a)*** The main effect of time in the cue-reactivity LME ((Drug Successful NoGo+Drug Successful Go) or (Neutral Successful NoGo+Neutral Successful Go)). ***(b)*** The main effect of time in the response inhibition LME ((Drug Successful NoGo+Neutral Successful NoGo) or (Drug Successful Go + Neutral Successful Go)). ***(c)*** The main effect of time in the methamphetamine-related response inhibition LME ((Drug Successful NoGo>Drug Successful Go) or (Neutral Successful NoGo>Neutral Successful Go)). (1) FDR corrected p-value<0.001. (2) FDR corrected p-value<0.05.

Linear Mixed Effect Models were analysed and visualized using R software version 3.6.2 (https://www.r-project.org/).

Abbreviation: SFG: superior frontal gyrus, MFG: middle frontal gyrus, IFG: inferior frontal gyrus, OrG: orbital gyrus, PrG: precentral gyrus, PCL: paracentral lobule, STG: superior temporal Gyrus, MTG: middle temporal gyrus, ITG: inferior temporal gyrus, FuG: fusiform gyrus, PhG: parahippocampal gyrus, pSTS:: posterior superior temporal sulcus, SPL: superior parietal lobule, IPL: inferior parietal lobule, Pcun: precuneus, PoG: postcentral gyrus, INS: insular gyrus, CG: cingulate gyrus, MVOcC: medioventral occipital cortex, LOcC: lateral occipital cortex, Amyg: amygdala, Hipp: hippocampus, BG: basal ganglia, Tha: thalamus

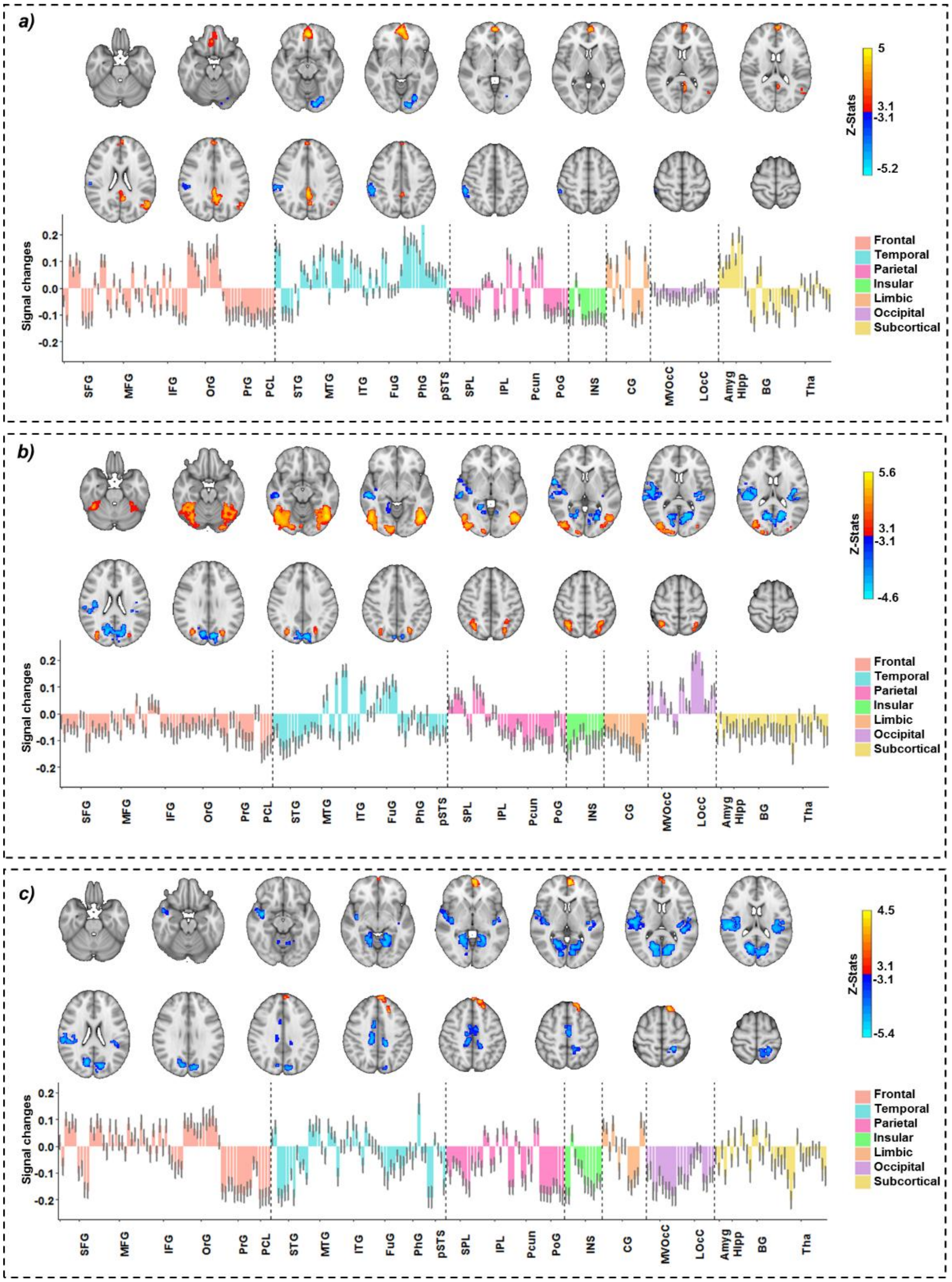

Supplementary Figure S3. Temporal behavior of the cue-reactivity contrast ((Drug Successful NoGo+Drug Successful Go) > (Neutral Successful NoGo + Neutral Successful Go))**.** Brain activation maps (Z-threshold > 3.1, alpha corrected *p*-value<0.001) and changes in brain activation in Brainnetome (BNA) regions. The colored bars show mean parameter estimates, and the error bars show the standard error of z-statistic values across 53 methamphetamine use disorders (MUD). ***(a)*** Window one. ***(b)*** Window two. ***(c)*** Window three.

Axial brain views were visualized using FSLeyes, the FSL image viewer (https://open.win.ox.ac.uk/pages/fsl/fsleyes/fsleyes/userdoc/install.html). The graphs were conducted and visualized using R software version 3.6.2 (https://www.r-project.org/).

Abbreviation: SFG: superior frontal gyrus, MFG: middle frontal gyrus, IFG: inferior frontal gyrus, OrG: orbital gyrus, PrG: precentral gyrus, PCL: paracentral lobule, STG: superior temporal Gyrus, MTG: middle temporal gyrus, ITG: inferior temporal gyrus, FuG: fusiform gyrus, PhG: parahippocampal gyrus, pSTS: posterior superior temporal sulcus, SPL: superior parietal lobule, IPL: inferior parietal lobule, Pcun: precuneus, PoG: postcentral gyrus, INS: insular gyrus, CG: cingulate gyrus, MVOcC: medioventral occipital cortex, LOcC: lateral occipital cortex, Amyg: amygdala, Hipp: hippocampus, BG: basal ganglia, Tha: thalamus.

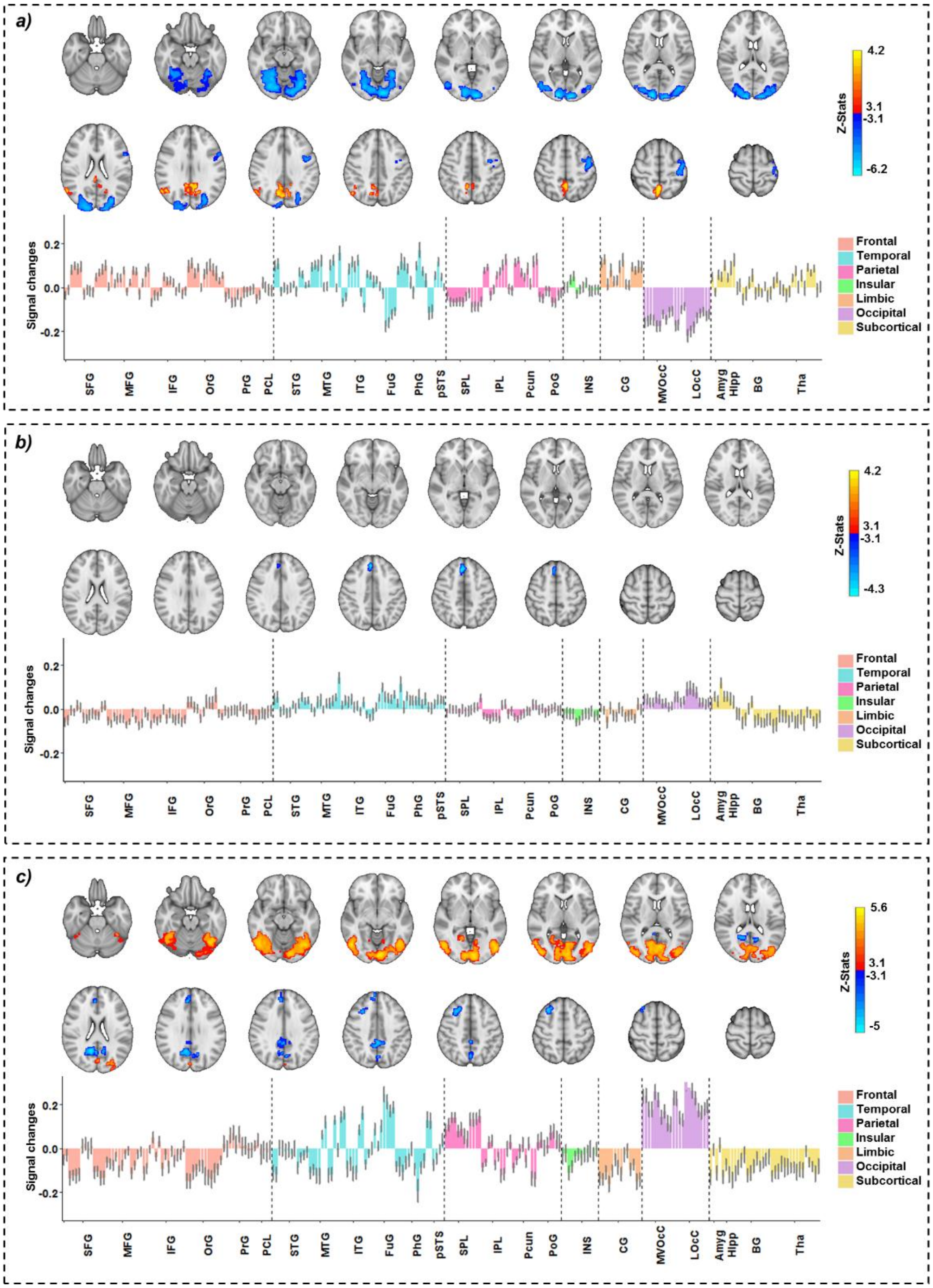

Supplementary Figure S4. Temporal behavior of the response inhibition contrast (Drug Successful NoGo +Neutral Successful Go) > (DrugSuccessful Go + Neutral Successful Go). Brain activation maps (Z-threshold > 3.1, alpha corrected p-value<0.001) and changes in brain activation in Brainnetome (BNA) regions. The colored bars show mean parameter estimates, and the error bars show the standard error of z-statistic values across 53 methamphetamine use disorders (MUD). **(a)** Window one. **(b)** Window two. **(c)** Window three.

Axial brain views were visualized using FSLeyes, the FSL image viewer (https://open.win.ox.ac.uk/pages/fsl/fsleyes/fsleyes/userdoc/install.html). The graphs were conducted and visualized using R software version 3.6.2 (https://www.r-project.org/).

Abbreviation: SFG: superior frontal gyrus, MFG: middle frontal gyrus, IFG: inferior frontal gyrus, OrG: orbital gyrus, PrG: precentral gyrus, PCL: paracentral lobule, STG: superior temporal Gyrus, MTG: middle temporal gyrus, ITG: inferior temporal gyrus, FuG: fusiform gyrus, PhG: parahippocampal gyrus, pSTS:: posterior superior temporal sulcus, SPL: superior parietal lobule, IPL: inferior parietal lobule, Pcun: precuneus, PoG: postcentral gyrus, INS: insular gyrus, CG: cingulate gyrus, MVOcC: medioventral occipital cortex, LOcC: lateral occipital cortex, Amyg: amygdala, Hipp: hippocampus, BG: basal ganglia, Tha: thalamus.

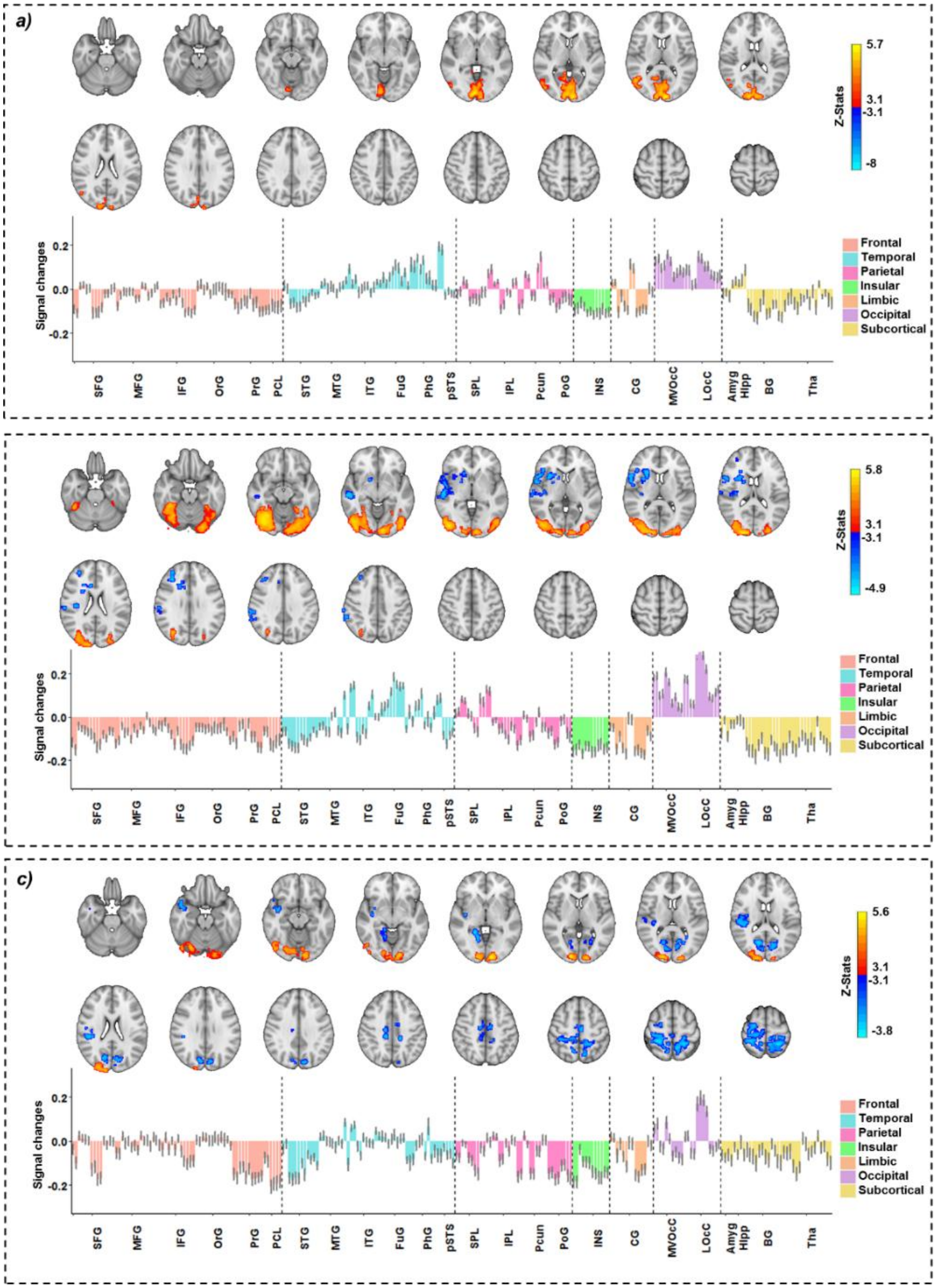

Supplementary Figure S5. Temporal behavior of the methamphetamine-related response inhibition contrast (Drug Successful NoGo - Drug Successful Go) > (Neutral Successful NoGo - Neutral Successful Go). Brain activation maps (Z-threshold > 3.1, alpha corrected p-value<0.001) and changes in brain activation in brainnetome (BNA) regions. The colored bars show mean parameter estimates and the error bars show the standard error of z-statistic values across 53 MUDs. **(a)** Window one. **(b)** Window two. **(c)** Window three.
Axial brain views were visualized using FSLeyes, the FSL image viewer (https://open.win.ox.ac.uk/pages/fsl/fsleyes/fsleyes/userdoc/install.html). The graphs were conducted and visualized using R software version 3.6.2 (https://www.r-project.org/).
Abbreviation: SFG: superior frontal gyrus, MFG: middle frontal gyrus, IFG: inferior frontal gyrus, OrG: orbital gyrus, PrG: precentral gyrus, PCL: paracentral lobule, STG: superior temporal Gyrus, MTG: middle temporal gyrus, ITG: inferior temporal gyrus, FuG: fusiform gyrus, PhG: parahippocampal gyrus, Psts: posterior superior temporal sulcus, SPL: superior parietal lobule, IPL: inferior parietal lobule, Pcun: precuneus, PoG: postcentral gyrus, INS: insular gyrus, CG: cingulate gyrus, MVOcC: medioventral occipital cortex, LOcC: lateral occipital cortex, Amyg: amygdala, Hipp: hippocampus, BG: basal ganglia, Tha: thalamus.

Supplementary Tables

Supplementary Table S1: Regions with significant condition by time interactions in the response inhibition linear mixed effects (LME) models. All *p*-values are FDR-corrected. FDR p-value threshold: 0.001.

Abbreviation: β: Beta value, SE: Standard Error, R: Right, L: Left, SFG: superior frontal gyrus, MFG: middle frontal gyrus, OrG: orbital gyrus, STG: superior temporal gyrus, MTG: middle temporal gyrus, aSTS: anterior superior temporal sulcus, ITG: inferior temporal gyrus, FuG: fusiform gyrus, PhG: parahippocampal gyrus, pSTS: posterior superior temporal sulcus, SPL: superior parietal lobule, IPL: Inferior Parietal Lobule, Pcun: precuneus, CG: cingulate gyrus, MVOcC: medioventral occipital cortex, LOcC: lateral Occipital Cortex, Amyg: amygdala, Hipp: hippocampus

Response Inhibition
(Drug Successful NoGo + Neutral Successful NoGo) > (Drug Successful Go + Neutral Successful Go)

	Condition				Time				Condition : Time			
**Location in BNA**	**β**	**SE**	**t-value**	***P*-value**	**β**	**SE**	**t-value**	***P*-value**	**β**	**SE**	**t-value**	***P*-value**
**L-SFG (dorsolateral)**	-0.17	0.03	-5.25	0.00005	-0.07	0.03	-2.54	1	0.25	0.04	6.02	0.000001
**R-SFG (dorsolateral)**	-0.17	0.03	-6.30	0.0000002	-0.08	0.02	-3.24	0.2521	0.24	0.03	7.07	0
**L-SFG (lateral)**	-0.19	0.03	-5.73	0.000004	-0.08	0.03	-2.6	1	0.24	0.04	5.83	0.000003
**R-SFG (lateral)**	-0.19	0.03	-6.32	0.0000002	-0.08	0.03	-2.99	0.5158	0.24	0.04	6.38	0.0000002
**L-SFG (medial area 9)**	-0.11	0.03	-3.96	0.0122	-0.05	0.02	-2.02	1	0.17	0.03	5.17	0.00007
**R-SFG (medial area 9)**	-0.12	0.03	-4.94	0.0002	-0.06	0.02	-2.74	1	0.18	0.03	5.76	0.000004
**L-SFG (medial area 10)**	-0.21	0.03	-6.03	0.000001	-0.08	0.03	-2.72	1	0.25	0.04	6.07	0.0000008
**R- SFG (medial area 10)**	-0.22	0.03	-6.85	0	-0.09	0.03	-3.21	0.2732	0.27	0.04	6.63	0
**L-MFG (area 46)**	-0.10	0.03	-3.822	0.0669	-0.04	0.02	-1.91	1	0.17	0.03	5.31	0.00004
**R-MFG (area 46)**	-0.11	0.02	-4.92	0.0002	-0.06	0.02	-2.99	0.5158	0.15	0.03	5.21	0.00006
**L-MFG (ventrolateral)**	-0.14	0.03	-5.07	0.0001	-0.06	0.02	-2.49	1	0.21	0.03	6.16	0.0000005
**R-MFG (ventrolateral)**	-0.09	0.02	-3.96	0.0125	-0.05	0.02	-2.51	1	0.15	0.03	5.56	0.00001
**L-MFG (lateral)**	-0.15	0.03	-4.94	0.0002	-0.06	0.03	-2.22	1	0.21	0.04	5.72	0.000005
**R-MFG (lateral)**	-0.13	0.02	-5.51	0.00001	-0.06	0.02	-2.91	0.6489	0.19	0.03	6.27	0.0000003
**L-OrG (medial)**	-0.13	0.02	-5.51	0.00001	-0.09	0.03	-2.92	0.6321	0.29	0.05	6.12	0.0000006
**R-OrG (medial)**	-0.25	0.04	-6.98	0	-0.11	0.03	-3.48	0.1166	0.30	0.04	6.78	0
**L-OrG (orbital)**	-0.16	0.03	-4.78	0.0005	-0.06	0.03	-2.01	1	0.20	0.04	4.99	0.0002
**R-OrG (orbital)**	-0.15	0.03	-5.63	0.000009	-0.07	0.02	-2.82	0.8161	0.20	0.03	6.03	0.000001
**L-OrG (medial)**	-0.22	0.04	-5.63	0.000008	-0.08	0.03	-2.26	1	0.26	0.05	5.23	0.00006
**R-OrG (medial)**	-0.23	0.04	-6.48	0	-0.09	0.03	-2.88	0.7103	0.27	0.04	6.12	0.0000006
**L-OrG (area 13)**	-0.21	0.04	-5.51	0.00002	-0.08	0.03	-2.52	1	0.26	0.05	5.58	0.00001
**L-STG (medial)**	-0.19	0.03	-5.75	0.000004	-0.09	0.03	-3.24	0.2499	0.23	0.04	5.50	0.00002
**R-STG (medial)**	-0.24	0.04	-6.83	0	-0.09	0.03	-3.19	0.2846	0.25	0.04	5.82	0.000003
**L-STG (lateral)**	-0.18	0.03	-6.52	0	-0.08	0.02	-3.56	0.0894	0.17	0.03	5.17	0.00007
**L-MTG (caudal)**	-0.17	0.03	-5.79	0.000003	-0.07	0.03	-2.78	0.9296	0.23	0.04	6.26	0.0000003
**R-MTG (caudal)**	-0.15	0.03	-5.46	0.00002	-0.06	0.02	-2.78	0.9296	0.21	0.03	6.46	0.0000001
**L-MTG (rostral)**	-0.22	0.04	-6.27	0.0000003	-0.09	0.03	-2.87	0.7283	0.25	0.04	5.93	0.000002
**R-MTG (rostral)**	-0.24	0.04	-6.70	0	-0.10	0.03	-3.34	0.1819	0.27	0.04	6.13	0.0000006

**R-MTG (dorsolateral)**	0.06	0.03	2.20	1	0.03	0.02	1.03	1	-0.17	0.04	-4.93	0.0002
**L-aSTS**	-0.21	0.03	-6.36	0.0000002	-0.09	0.03	-3.02	0.4863	0.25	0.04	6.06	0.0000008
**R-aSTS**	-0.24	0.03	-7.50	0	-0.10	0.03	-3.73	0.0481	0.26	0.04	6.61	0
**L-ITG (intermediate ventral)**	0.12	0.02	5.05	0.0001	0.04	0.02	1.89	1	-0.17	0.03	-5.71	0.000005
**L-ITG (extreme lateroventral)**	0.22	0.03	8.41	0	0.09	0.02	3.72	0.0505	-0.25	0.03	-7.71	0
**R-ITG (extreme lateroventral)**	0.20	0.03	7.08	0	0.08	0.02	3.09	0.3968	-0.25	0.03	-7.18	0
**L-ITG (rostral)**	-0.19	0.04	-5.30	0.00004	-0.07	0.03	-2.39	1	0.20	0.04	4.62	0.0009
**R-ITG (rostral)**	-0.23	0.03	-7.76	0	-0.10	0.03	-3.77	0.0415	0.18	0.04	5.17	0.00007
**L-ITG (intermediate lateral)**	-0.19	0.03	-5.65	0.000007	-0.07	0.03	-2.56	1	0.24	0.04	5.87	0.000002
**R-ITG (intermediate lateral)**	-0.21	0.03	-6.92	0	-0.09	0.03	-3.42	0.1434	0.25	0.04	6.51	0
**L-ITG (ventrolateral)**	0.12	0.02	4.95	0.0002	0.04	0.02	1.73	1	-0.15	0.03	-4.93	0.0002
**R-ITG (ventrolateral)**	0.19	0.03	6.33	0.0000002	0.09	0.03	3.65	0.0639	-0.29	0.04	-7.99	0
**L-ITG (caudolateral)**	-0.08	0.02	-3.08	0.2552	-0.03	0.02	-1.49	1	0.15	0.03	5.03	0.0001
**L-FuG (medioventral)**	0.26	0.03	7.55	0	0.17	0.03	5.53	0.00001	-0.43	0.04	-10.12	0
**R-FuG (medioventral)**	0.23	0.03	6.93	0	0.15	0.03	5.17	0.0001	-0.38	0.04	-9.51	0
**L-FuG (lateroventral)**	0.22	0.03	7.87	0	0.12	0.02	4.69	0.0010	-0.31	0.03	-8.95	0
**R-FuG (lateroventral)**	0.18	0.03	6.34	0.0000002	0.11	0.02	4.38	0.0038	-0.31	0.04	-8.88	0
**L-PhG (rostral)**	-0.13	0.03	-4.85	0.0003	-0.09	0.02	-3.77	0.0415	0.16	0.03	4.72	0.0005
**L-PhG (caudal)**	-0.11	0.04	-3.03	0.2952	-0.12	0.03	-3.85	0.0313	0.21	0.04	4.73	0.0005
**L-PhG (entorhinal)**	-0.15	0.03	-5.18	0.00008	-0.09	0.02	-3.84	0.0321	0.19	0.03	5.52	0.00001
**R-PhG (entorhinal)**	-0.33	0.05	-6.25	0.0000003	-0.17	0.05	-3.66	0.0627	0.39	0.06	6.03	0.000001
**L-PhG (temporal insular cortex)**	-0.12	0.03	-4.57	0.0011	-0.09	0.02	-3.84	0.0327	0.16	0.03	4.77	0.0004
**L-PhG (medial PPHC)**	0.05	0.04	1.25	1	0.06	0.03	1.97	1	-0.24	0.04	-5.46	0.00002
**R-PhG (medial PPHC)**	0.08	0.04	2.16	1	0.05	0.03	1.64	1	-0.24	0.04	-5.50	0.00001
**R-pSTS (rpSTS)**	-0.22	0.03	-8.24	0	-0.09	0.02	-3.86	0.0308	0.15	0.03	4.72	0.0005
**L-SPL (rostral)**	0.07	0.03	2.66	0.7901	0.03	0.02	1.13	1	-0.16	0.03	-4.85	0.0003
**R-SPL (rostral)**	0.08	0.03	3.04	0.2871	0.05	0.02	2.24	1	-0.20	0.03	-5.81	0.00003
**L-SPL (caudal)**	0.13	0.03	5.09	0.0001	0.07	0.02	3.01	0.5015	-0.24	0.03	-7.51	0
**R-SPL (caudal)**	0.14	0.03	5.27	0.00005	0.07	0.02	2.87	0.7286	-0.25	0.03	-7.41	0
**L-SPL (lateral)**	0.13	0.03	4.26	0.0040	0.05	0.03	2.12	1	-0.17	0.04	-4.67	0.0006
**R-SPL (lateral)**	0.09	0.03	3.24	0.1.591	0.06	0.03	2.29	1	-0.17	0.04	-4.94	0.0002
**L-SPL (intraparietal)**	0.09	0.03	3.24	0.1591	0.09	0.03	3.29	0.2208	-0.26	0.04	-6.84	0
**R-SPL (intraparietal)**	0.17	0.03	5.57	0.00001	0.08	0.03	3.21	0.2743	-0.25	0.04	-6.79	0
**L-IPL (caudal PGp)**	0.15	0.03	5.61	0.000009	0.08	0.02	3.26	0.2380	-0.23	0.03	-7.27	0
**R-IPL (caudal PGp)**	0.08	0.03	3.01	0.3082	0.06	0.02	2.44	1	-0.23	0.03	-6.76	0
**L-IPL (rostrodorsal)**	-0.11	0.02	-4.55	0.0012	-0.05	0.02	-2.32	1	0.18	0.03	6.31	0.0000002
**R-IPL (rostrodorsal)**	-0.09	0.02	-4.64	0.0008	-0.05	0.02	-3.18	0.3037	0.16	0.02	6.41	0.0000001
**L-IPL (caudal PFm)**	-0.09	0.02	-3.98	0.0114	-0.04	0.02	-1.86	1	0.13	0.03	4.69	0.0006
**L-IPL (rostroventral)**	-0.22	0.03	-6.63	0	-0.09	0.03	-3.15	0.3268	0.25	0.04	6.41	0.0000001
**L-IPL (rostroventral)**	-0.23	0.29	-8.05	0	-0.10	0.02	-4.09	0.0129	0.26	0.04	7.39	0

**L-Pcun (medial)**	-0.18	0.03	-6.96	0	-0.09	0.02	-4.36	0.0042	0.20	0.03	6.38	0.0000001
**R-Pcun (medial)**	-0.15	0.02	-6.62	0	-0.09	0.02	-4.93	0.0003	0.16	0.03	5.64	0.000007
**L-Pcun (Lc1)**	-0.25	0.03	-7.20	0	-0.10	0.03	-3.41	0.1477	0.29	0.04	6.84	0
**R-Pcun (Lc1)**	-0.26	0.03	-7.77	0	-0.11	0.03	-3.90	0.0259	0.30	0.04	7.34	0
**L-CG (dorsal)**	-0.22	0.03	-6.82	0	-0.09	0.03	-3.39	0.1566	0.28	0.04	7.02	0
**R-CG (dorsal)**	-0.23	0.03	-7.50	0	-0.11	0.03	-4.03	0.0160	0.29	0.04	7.80	0
**R-CG (rostroventral)**	-0.23	0.04	-6.12	0.0000006	-0.10	0.03	-3.11	0.3734	0.27	0.05	5.97	0.000001
**L-CG (ventral)**	-0.27	0.04	-7.71	0	-0.09	0.03	-3.27	0.2291	0.21	0.04	4.78	0.0004
**R-CG (ventral)**	-0.28	0.03	-8.15	0	-0.13	0.03	-4.43	0.0031	0.26	0.04	6.18	0.0000004
**L-CG (caudal)**	-0.25	0.03	-7.85	0	-0.11	0.03	-3.84	0.0321	0.18	0.04	4.62	0.0008
**L-CG (subgenual)**	-0.24	0.04	-6.51	0	-0.09	0.03	-2.99	0.5157	0.29	0.05	6.29	0.0000002
**R-CG (subgenual)**	-0.22	0.03	-7.04	0	-0.09	0.03	-3.46	0.1259	0.26	0.04	6.73	0
**L-MVOcC (caudal lingual gyrus)**	0.24	0.03	7.31	0	0.15	0.03	5.31	0.00005	-0.41	0.04	-10.14	0
**R-MVOcC (caudal lingual gyrus)**	0.25	0.03	7.49	0	0.15	0.03	5.14	0.0001	-0.41	0.04	-10.13	0
**L-MVOcC (rostral cuneus gyrus)**	0.15	0.03	4.91	0.0003	0.12	0.03	4.46	0.0028	-0.33	0.04	-8.59	0
**R-MVOcC (rostral cuneus gyrus)**	0.15	0.03	4.74	0.0005	0.12	0.03	4.46	0.0028	-0.32	0.04	-8.30	0
**L-MVOcC (caudal cuneus gyrus)**	0.27	0.04	7.51	0	0.17	0.03	5.53	0.00002	-0.45	0.04	-10.21	0
**R-MVOcC (caudal cuneus gyrus)**	0.23	0.03	6.67	0	0.16	0.03	5.33	0.00005	-0.40	0.04	-9.63	0
**L-MVOcC (rostral lingual gyrus)**	0.11	0.03	3.73	0.0285	0.10	0.03	3.85	0.0318	-0.26	0.04	-7.02	0
**R-MVOcC (rostral lingual gyrus)**	0.16	0.03	5.25	0.00005	0.12	0.03	4.48	0.0025	-0.32	0.04	-8.24	0
**L-MVOcC (parietooccipital)**	0.07	0.03	2.24	1	0.08	0.02	3.01	0.5014	-0.22	0.04	-5.87	0.000002
**R-MVOcC (parietooccipital)**	0.06	0.03	1.98	1	0.07	0.03	2.85	0.7610	-0.21	0.04	-5.66	0.000006
**L-LOcC (middle occipital gyrus)**	0.26	0.03	7.84	0	0.17	0.03	5.71	0.000007	-0.41	0.04	-10.06	0
**R-LOcC (middle occipital gyrus)**	0.26	0.03	7.78	0	0.16	0.03	5.71	0.000007	-0.41	0.04	-10.09	0
**L-LOcC (V5/MT+)**	0.13	0.03	4.61	0.0009	0.09	0.02	3.94	0.0227	-0.25	0.03	-7.34	0
**R-LOcC (V5/MT+)**	0.10	0.03	3.92	0.0142	0.08	0.02	3.62	0.0709	-0.22	0.03	-6.83	0
**L-LOcC (occipital polar cortex)**	0.40	0.04	9.62	0	0.23	0.04	6.37	0.0000002	-0.53	0.05	-10.38	0
**R-LOcC (occipital polar cortex)**	0.42	0.04	9.82	0	0.22	0.04	6.06	0.000001	-0.49	0.05	-9.52	0
**L-LOcC (inferior occipital gyrus)**	0.40	0.04	10.35	0	0.19	0.03	5.79	0.000004	-0.47	0.05	-9.84	0
**R-LOcC (inferior occipital gyrus)**	0.29	0.03	8.68	0	0.15	0.03	5.28	0.00006	-0.39	0.04	-9.54	0
**L-LOcC (medial superior occipital)**	0.14	0.03	4.66	0.0007	0.10	0.03	4.12	0.0113	-0.30	0.04	-8.45	0
**R-LOcC (medial superior occipital)**	0.12	0.03	4.24	0.0043	0.09	0.02	3.91	0.0253	-0.28	0.04	-8.04	0
**L-LOcC (lateral superior occipital)**	0.19	0.03	6.32	0.0000002	0.12	0.03	4.64	0.0013	-0.32	0.04	-8.86	0
**R-LOcC (lateral superior occipital)**	0.19	0.03	6.33	0.0000002	0.12	0.03	4.5	0.0018	-0.33	0.04	-9.06	0
**L-Amyg (medial amygdala)**	-0.13	0.03	-4.03	0.0094	-0.09	0.03	-3.08	0.4121	0.21	0.04	5.47	0.00002
**L-Amyg (lateral amygdala)**	-0.14	0.03	-4.34	0.0029	-0.08	0.03	-3.00	0.5033	0.19	0.04	4.78	0.0004
**L-Hipp (rostral hippocampus)**	-0.22	0.03	-6.34	0.0000002	-0.11	0.03	-3.54	0.0939	0.26	0.04	6.04	0.0000009
**L-Hipp (caudal hippocampus)**	-0.18	0.04	-4.56	0.0012	-0.10	0.03	-3.04	0.4632	0.23	0.05	4.85	0.0003
**R-Hipp (caudal hippocampus)**	-0.18	0.04	-5.03	0.00015	-0.14	0.03	-4.25	0.0065	0.28	0.04	6.20	0.0000004

Supplementary Table S2: The Correlation between cue reactivity activation slopes and behavioral and clinical data, as well as the correlation between the subjects with positive and negative cue reactivity slopes respectively and behavioral and clinical data.

**Behavioral Variable**	**Brainnetome ROIs**	**Beta**	**P-value**	
			**Uncorrected**	**Corrected**
Duration of Abuse	left Precuneus	0.282	0.0407	0.2852
Omission Error	Right lateral Superior Temporal Gyrus	-0.362	0.0076	0.1938
	Left rostral Superior Temporal Gyrus	-0.386	0.0042	0.0214
	Right rostral Superior Temporal Gyrus	-0.375	0.0055	0.0221
	left Precuneus	-0.414	0.0020	0.0133
	Right medial Amygdala	-0.334	0.0142	0.0427
	Right lateral Amygdala	-0.281	0.0410	0.0821
	Right rostral Hippocampus	-0.416	0.0019	0.0133
Total Reaction Time	Right lateral Superior Temporal Gyrus	-0.320	0.0100	0.0600
	left Precuneus	-0.290	0.0300	0.2100
	Right rostral Hippocampus	-0.290	0.0300	0.2100
**Positive Beta Values with Behavioral Variables**				
Pre VAS	Left Precuneus	0.329	0.0330	0.1130
Post Vas	Right lateral Superior Temporal Gyrus	0.311	0.0445	0.3444
Barrat Sum	Left Precuneus	0.313	0.0456	0.3194
Barrat Motor	Right intermediate Inferior Temporal Gyrus	0.314	0.0452	0.2716
	Left Precuneus	0.364	0.0190	0.1335
Number of Abuse days in last month	Right lateral Superior Temporal Gyrus	0.412	0.0066	0.0165
	Left lateral Superior Temporal Gyrus	4371	0.0047	0.0182
	Right rostral Superior Temporal Gyrus	0.448	0.0037	0.0182
	Right intermediate Inferior Temporal Gyrus	0.461	0.0024	0.0168
	Left Precuneus	0.444	0.0036	0.0182
	Right medial Amygdala	0.399	0.0080	0.0182
	Right lateral Amygdala	0.451	0.0030	0.0182
	Right rostral Hippocampus	0.338	0.0350	0.1220
Total risky behavior	Right intermediate Inferior Temporal Gyrus	0.439	0.0040	0.0284
	Left Precuneus	0.352	0.0238	0.1430
Dosage of Meth Use	Right intermediate Inferior Temporal Gyrus	-0.405	0.0084	0.0594
**Negative Beta Values with Behavioral Variables**				
Pre VAS	Left lateral Superior Temporal Gyrus	0.535	0.0500	0.4173
Barrat Sum	Left lateral Superior Temporal Gyrus	0.623	0.0228	0.1372
	Right rostral Superior Temporal Gyrus	0.685	0.0097	0.0684
	Right rostral Hippocampus	0.563	0.0358	0.2152
Number of Abuse days in last month	Right lateral Superior Temporal Gyrus	0.619	0.0422	0.2147
	Left Precuneus	0.632	0.0272	0.1636
	lateral Amygdala	0.595	0.0409	0.2047
	Right rostral Hippocampus	0.683	0.0070	0.0491
Total Drug cost	Right rostral Superior Temporal Gyrus	0.654	0.0152	0.1065
Total Risky Behavior	Right lateral Superior Temporal Gyrus	0.621	0.0411	0.4117
	Right rostral Superior Temporal Gyrus	0.654	0.0152	0.1065

Supplementary Table S3: The Correlation between response inhibition activation slopes and behavioral and clinical data, as well as the correlation between the subjects with positive and negative response inhibition slopes respectively and behavioral and clinical data.

**Behavioral Variable**	**Brainnetome ROIs**	**Beta**	**P-value**	
			**Uncorrected**	**Corrected**
Total drug cost in the last month	Right lateral Superior Temporal Gyrus	0.546	0.0190	0.0390
Total Reaction Time	Right lateral Superior Temporal Gyrus	0.277	0.0440	0.1610
**Positive Beta Values with Behavioral Variables**				
Barrat Sum	Right lateral Superior Temporal Gyrus	0.383	0.0227	0.2145
Barrat Motor	Right lateral Superior Temporal Gyrus	0.364	0.0314	0.1308
	Left Precuneus	0.344	0.0319	0.1279
Pre VAS	Right lateral Superior Temporal Gyrus	0.358	0.0345	0.1245
	Right intermediate ventral Superior Temporal Gyrus	0.370	0.0340	0.1361
Number of Abuse days in last month	Right lateral Superior Temporal Gyrus	0.452	0.0062	0.0016
	Right intermediate ventral Superior Temporal Gyrus	0.482	0.0044	0.0176
	Left Precuneus	0.415	0.0086	0.0258
Post VAS	Right lateral Superior Temporal Gyrus	0.318	0.0622	0.0622
	Right intermediate ventral Superior Temporal Gyrus	0.362	0.0384	0.1536
Total Risky Behavior	Right lateral Superior Temporal Gyrus	0.482	0.0033	0.0133
	Right intermediate ventral Superior Temporal Gyrus	0.376	0.0309	0.1238
Total Reaction Time	Left Precuneus	0.312	0.0523	0.2095
Dosage of Meth Use	Right intermediate ventral Superior Temporal Gyrus	-0.380	0.0287	0.1151
**Negative Beta Values with Behavioral Variables**				
Number of Abuse days in last month	Right lateral Superior Temporal Gyrus	0.546	0.0190	0.0390
	Right intermediate ventral Superior Temporal Gyrus	0.466	0.0382	0.0554
	Left Parahippocampal Gyrus	0.507	0.0007	0.0026
	Right Parahippocampal Gyrus	0.515	0.0006	0.0027
	Left Precuneus	0.585	0.0277	0.0554
Total Risky Behavior	Left Parahippocampal Gyrus	0.327	0.0364	0.1174
	Right Parahippocampal Gyrus	0.311	0.0505	0.2750
	Left Precuneus	0.580	0.0293	0.1174
Dosage of Meth Use	Right lateral Superior Temporal Gyrus	-0.512	0.0296	0.1296

Supplementary Table S4: The Correlation between methamphetamine-related response inhibition activation slopes and behavioral and clinical data, as well as the correlation between the subjects with positive and negative methamphetamine-related response inhibition slopes respectively and behavioral and clinical data.

**Behavioral Variable**	**Brainnetome ROIs**	**Beta**	**P-value**	
			**Uncorrected**	**Corrected**
**Positive Beta Values with Behavioral Variables**				
Number of Abuse days in last month	Left Parahippocampal Gyrus	0.505	0.0010	0.0020
	Right Parahippocampal Gyrus	0.505	0.0010	0.0020
	Right Precuneus	0.512	0.0009	0.0019
Total Risky Behavior	Left Parahippocampal Gyrus	0.329	0.0402	0.0805
	Right Parahippocampal Gyrus	0.329	0.0402	0.0805
**Negative Beta Values with Behavioral Variables**				
Total Risky Behavior	Right Precuneus	0.586	0.0216	0.0432
Dosage of Meth Use	Right Precuneus	-0.583	0.022	0.045
